# Investigating composite electrode materials of metal oxides for advanced energy storage applications

**DOI:** 10.1186/s40580-024-00437-2

**Published:** 2024-07-30

**Authors:** Parthiban Pazhamalai, Vignesh Krishnan, Mohamed Sadiq Mohamed Saleem, Sang-Jae Kim, Hye-Won Seo

**Affiliations:** 1 Nanomaterials & System Laboratory, Major of Mechatronics Engineering, Faculty of Applied Energy System, Jeju National University, Jeju, 63243 South Korea; 2 Research Institute of New Energy Industry (RINEI), Jeju National University, Jeju, 63243 South Korea; 3 Nanomaterials & System Lab, Major of Mechanical System Engineering, College of Engineering, Jeju National University, Jeju, 63243 South Korea; 4 Department of Physics, Jeju National University, Jeju, 63243 South Korea

## Abstract

Electrochemical energy systems mark a pivotal advancement in the energy sector, delivering substantial improvements over conventional systems. Yet, a major challenge remains the deficiency in storage technology to effectively retain the energy produced. Amongst these are batteries and supercapacitors, renowned for their versatility and efficiency, which depend heavily on the quality of their electrode materials. Metal oxide composites, in particular, have emerged as highly promising due to the synergistic effects that significantly enhance their functionality and efficiency beyond individual components. This review explores the application of metal oxide composites in the electrodes of batteries and SCs, focusing on various material perspectives and synthesis methodologies, including exfoliation and hydrothermal/solvothermal processes. It also examines how these methods influence device performance. Furthermore, the review confronts the challenges and charts future directions for metal oxide composite-based energy storage systems, critically evaluating aspects such as scalability of synthesis, cost-effectiveness, environmental sustainability, and integration with advanced nanomaterials and electrolytes. These factors are crucial for advancing next-generation energy storage technologies, striving to enhance performance while upholding sustainability and economic viability.

## Introduction

The development of diverse energy systems, encompassing energy conversion and storage, has been strategically advanced to address the increasing global demand for power and effectively mitigate the energy crisis. Various renewable energy systems are available that transform alternative energy sources, such as solar, wind, hydroelectric, and thermal into electricity—a technological evolution that marks this era [1, 2]. However, the deficiency in storage technology for retaining produced energy remains a significant hurdle, as its efficiency has not yet reached the level required for optimal energy delivery [3, 4].

In energy storage research, batteries and supercapacitors stand out for their unique contributions to sustainable and efficient energy solutions. Batteries are crucial for applications requiring long-lasting power and high energy density in compact spaces. Supercapacitors, with their rapid charging and high-power density, excel in situations needing quick energy bursts. Their unparalleled cycle life also offers reliability unmatched by batteries. The distinct advantages of each technology highlight their complementary roles in enhancing energy storage systems, underscoring the importance in advancing sustainable energy technologies [5, 6]. Therefore, both the batteries and supercapacitors become the prime components for high energy densities for long-term storage and high power energy densities for short-term balancing [7]. Although fuel cells demonstrate relatively high energy conversion efficiency, they have a shorter lifespan and decreased durability [8].

The quest for optimized efficiency features designing and developing ingenious electrode materials for batteries and supercapacitors. The efficiency of these energy storage systems is intrinsically tied to the electrochemical characteristics and physical properties of their electrodes. In that regard, researchers have explored various materials with different configurations, including carbon/its derivatives, metal oxides, metal hydroxides, sulfides, selenides, MXenes, and their composites [9–12]. Metal oxides have emerged as compelling candidates for Energy Storage Systems (ESS) due to their comprehensive properties- flexibility, transparency, semi-conductivity, photosensitivity, and redox capabilities. Additionally, these boast abundant active sites, exceptional mechanical strength, a high specific surface area, and robust chemical stability, making them highly advantageous for various energy storage [13, 14]. The metal oxides has advantages over sulfides, phosphides, and carbides due to their superior chemical and thermal stability, essential for enduring performance in energy storage [15]. Metal oxides are safer, more environmentally friendly, and subject to stringent commercial regulations, making them ideal for widespread implementation. Furthermore, metal oxides are more abundant and cost-effective, contrasting with the more expensive and complex synthesis required for other compounds [15]. These attributes render metal oxides optimal for sustainable and economically viable energy storage solutions.

Given these advantages, metal oxides and their composite derivatives have increasingly been recognized as promising electrode materials, valued for their versatile functionalities [16]. Specific material properties and device functionalities unattainable with individual materials become achievable through composite configurations. The synergistic effects of these composites enhance electrode performance, making them excellent choices for high-efficiency energy systems and advancing sustainable technologies [14]. Prior reviews have illuminated these advantages: Lokhande et al. highlighted the improved conductivity, cycle stability, and capacitance achieved by combining metal oxides with carbon based materials [17]; Hussain et al. detailed the synergistic effects of incorporating graphene with transition metal oxides, noting that this combination significantly enhances the structural integrity and energy density of the composites, while the high electrical conductivity and extensive surface area of graphene contribute to increased charge storage capacity and accelerated charge/discharge cycles [18]; Wang et al. also demonstrated that embedding metal nanoparticles and their oxides into a porous carbon matrix enhances capacitance and cyclic stability. These nanoparticles impart pseudocapacitive properties to the matrix, significantly elevating the overall capacitance beyond the capabilities of carbon alone [19]; and Shaikh et al. recently explored how graphene composites, when combined with conducting polymers and MXenes, boost both conductivity and mechanical strength, underscoring the critical role of composite materials in supercapacitor advancements [20]. Despite these promising attributes, challenges still remain, such as achieving consistent distribution of metal oxides, preventing metal particle agglomeration, and ensuring long-term stability, which necessitates further research to stabilize these composites during repeated use [18, 19].

**Fig. 1:**
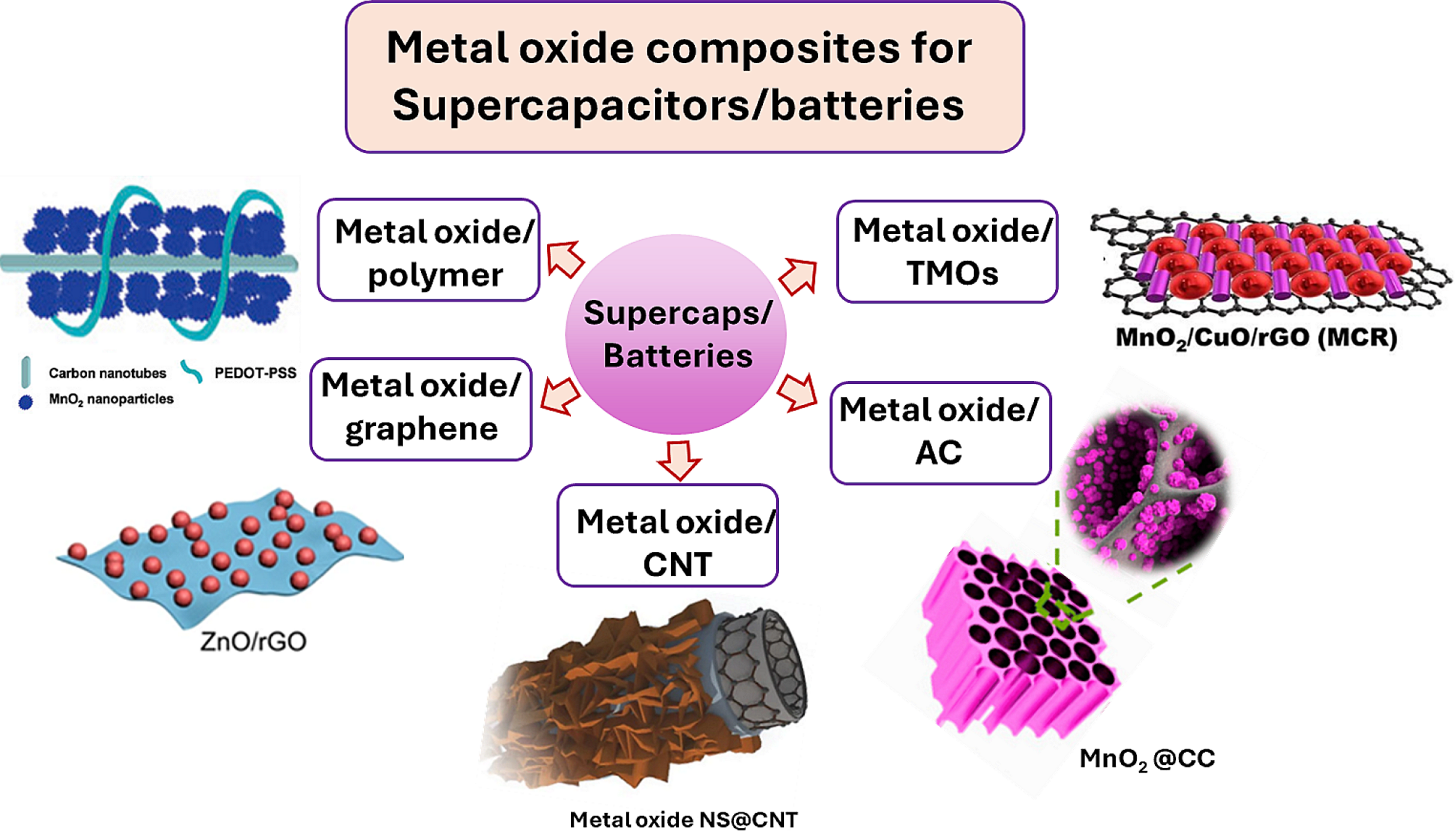
Schematic representation of the various types of metal composites based electrode materials for energy storage applications

This review delves into the use of metal oxides and their composites in energy storage systems, with a specific focus on electrodes for supercapacitors and batteries, as depicted in Fig. 1. It provides a detailed examination of various electrode configurations, aiming to offer a comprehensive understanding of their roles and potential for enhancing energy storage solutions, based on the most recent research findings. We address the challenges faced in development and implementation, evaluate the prospects for commercialization, and provide insights into future trends in this field. Our goal is to not only enrich academic knowledge but also to catalyze practical advancements in energy storage technologies.

## Methodologies involved in the preparation of metal oxide composites

### Top-down approach

Generally, the fabrication of metal oxide nanocomposites falls into two main categories: “top-down” and “bottom-up” synthesis [21]. The top-down approach involves reducing bulk structures to nanoscale dimensions through mechanical or chemical processes. However, this approach alone is not sufficient for creating nanocomposite materials. Rather, it serves to prepare individual templates, matrices, or nanostructures. Key techniques in top-down synthesis, such as exfoliation, mechanical milling, and lithography, are discussed in the following section. These methods are chosen for their relevance and represent some of the most common strategies in this approach [22].

#### Exfoliation

Exfoliation is a method for creating two-dimensional (2D) materials by physically or chemically peeling layers from bulk materials to produce atomically thin sheets [23]. The chemical (liquid) exfoliation method involves dispersing materials in solvents and utilizing sonication followed by centrifugation to isolate mono- or few-layer sheets. In contrast, physical (mechanical) exfoliation typically uses adhesive tape to peel off layers from bulk material, producing thin layers. This tape is repeatedly folded and unfolded, gradually reducing the material to the desired thickness. Originally used to isolate graphene, this technique allows for observing quantum properties in 2D materials. However, it faces challenges such as low yield, small flake sizes, and potential contamination, limiting its scalability. Despite these drawbacks, exfoliation remains crucial for research into the unique properties of 2D materials [24].

**Fig. 2:**
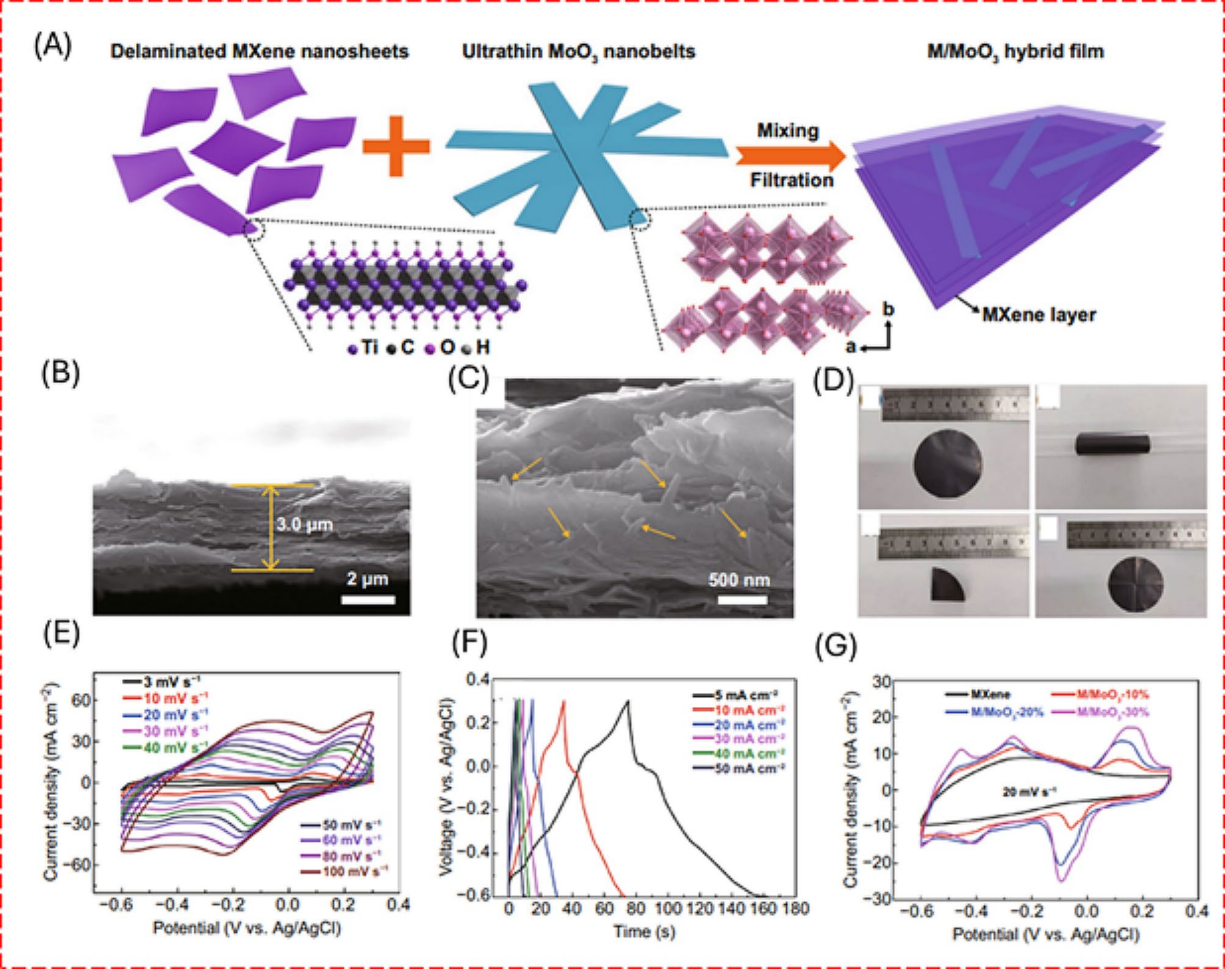
(**A**) Schematic illustration of the fabrication process of M/MoO_3_ hybrid flms (**B**, **C**) cross-sectional images (**D**) film under deformation of M/MoO_3_. (**E**) CV curves of the M/MoO_3_ electrode at various scan rates, (**F**) GCD profiles of M/MoO_3_ electrode at various current densities, (**G**) CV curves of pure MXene and composite electrodes at a scan rate of 20 mV s^− 1^. [30]

Exfoliation processes have proven to be an effective and formidable strategy for synthesizing a diverse array of materials, including graphene and graphene oxides (GO) derived from layered graphite and a variety of metal oxide nanosheets such as VO_2,_ V_2_O_5_, MnO_2_, MoO_3_, and SnO [25–28]. Additionally, this method has facilitated the production of novel 2D materials, silicenes, and MXenes [29]. Post-hydrothermal treatments are often employed to enhance the properties of these exfoliated materials further. Figure 2 shows the exfoliation and preparation of composites for the energy storage application. These treatments allow for advanced surface modifications, including doping, which can significantly improve the material functionalities [29, 30]. Significantly, the utility of exfoliation extends beyond mere synthesis; it also serves as a crucial surface treatment technique. However, exfoliating layered compounds with high layer charge density continues to pose a significant challenge. Furthermore, acquiring non-layered materials is particularly complex because the in-plane bonds are substantially stronger than van der Waals forces. As a result, mechanical and chemical exfoliation methods have consistently proven ineffective for these materials [29].

#### Mechanical milling

Mechanical milling is a prominent method within the top-down approach of nanomaterial synthesis, leveraging mechanical forces to reduce bulk materials to nanoscale particles [23, 31]. In this process, bulk materials are placed in a milling vessel along with milling balls or media, such as stainless steel balls or ceramic beads [32]. The milling vessel is then rotated, causing the balls to collide with the material, exerting mechanical impact and frictional forces. These interactions result in the gradual breakdown of the bulk material into smaller particles [33]. The intensity and duration of milling can be controlled to achieve the desired particle size distribution and properties. Factors such as milling time, milling speed, and the choice of milling media must be considered to optimize the process and avoid unintended structural changes or contamination in the synthesized nanoparticles.

A copper oxide-based composite in graphene with a less defective 2D network was synthesized using a ball milling technique, and its application was studied for hybrid supercapacitors (SCs) [34]. This work reports graphene produced through ball milling with D-sorbitol and utilized as an electrode material to boost the conductivity and electrochemical capabilities of copper oxide electrodes. Significant ball impacts are required to induce chemical transformations, resulting in structural stress, bond disintegration, and the generation of reactive radicals. This process creates elastic deformations and exposes reactive layers of atoms at the reactant interface, thereby promoting efficient reactions [35]. However, mechanical milling comes with several challenges. It can introduce impurities from the milling media and container, affecting product purity. The technique also produces a wide range of particle sizes, consumes considerable energy, and generates heat, which can alter material properties and may require cooling measures to manage [35].

#### Lithography

Lithography techniques are mainly used for precise patterning and etching materials at the nanoscale, enabling the creation of intricate nanostructures essential for various applications. Photolithography and electron beam lithography (EBL) are two main techniques for synthesizing nanomaterials with absolute precision [36]. Photolithography, a widely used method, involves using Visible or UV light to transfer patterns onto a substrate coated with a photosensitive material, typically a photoresist. On the other hand, in EBL, a focused beam of electrons is used to directly write patterns onto a substrate coated with an electron-sensitive resist material. EBL offers even greater resolution and flexibility in nanostructure fabrication than photolithography. The electron beam can be controlled with high precision, allowing for the creation of features as small as a few nanometers. Both photolithography and EBL play critical roles in advancing nanotechnology by enabling the fabrication of nanostructures with tailored properties and functionalities. Patterned 3D bio-inspired structures are reported using graphene-based PANI composite, and its SC performance was analyzed [37]. These conductive composites-based electrode materials enable flexible devices with enhanced ion–electrode transport, which is a significant advantage for an ideal electrode that outperforms energy storage applications [38].

### Bottom-up approach

Bottom-up synthesis provides substantial benefits by enabling precise control over the composition, morphology, and size of low-dimensional materials, facilitating the creation of highly customized materials. This method constructs nanomaterials typically via self-assembly or controlled growth processes. In this technique, the materials are assembled atom-by-atom or molecule-by-molecule, ensuring detailed structural configurations tailored to specific applications [39]. Common techniques in bottom-up synthesis include sol-gel synthesis, hydrothermal, solvothermal synthesis, chemical vapor depositions, and so on. Each method of approach has its advantages and limitations, and the choice between them depends on factors such as desired properties, scalability, and cost-effectiveness for a specific application, which is discussed in detail.

#### Sol-gel methods

The sol-gel process involves the conversion of a precursor solution (sol) into a gel-like network, followed by drying and annealing to form the desired nanomaterials [40]. By adjusting factors such as the precursors, catalysts, solvent, and drying duration/temperature, this method can be customized to suit specific needs. The versatility of the sol-gel process allows for the integration of organic and temperature-sensitive materials across a variety of applications. [21, 41]. However, the sol-gel method has its limitations, including significant shrinkage during drying and firing that can lead to cracking, lengthy processing times, and challenges in scaling up for industrial production. Additionally, the cost of raw materials and the use of hazardous chemicals can increase operational complexity and expenses. Despite these challenges, the sol-gel method remains a valuable tool for synthesizing a wide range of high-purity materials, provided that the processing and economic hurdles are effectively managed. For instance, the sol-gel process is employed to precisely synthesize high-quality metal oxide nanosheets, which are well-suited for battery applications due to their high specific surface area and optimal morphological characteristics. These nanosheets are prepared using a citrate-assisted sol-gel method, involving sequential steps of evaporation, drying, and calcination to achieve the desired properties [42].

The sol-gel process is also versatile, accommodating a range of metal oxides such as aluminum oxide, titanium dioxide, zinc oxide, and iron oxide, making it ideal for producing composites tailored to various applications [43]. This method ensures a uniform coating of CNTs with metal oxides, which is particularly vital for electronic and photonic applications where consistent behavior across the system is essential. Additionally, the sol-gel method may enhance the interface bonding between CNTs and metal oxides, significantly improving the mechanical strength and electrical conductivity of the composites. Such robust bonding, usually covalent, is crucial for applications demanding durable structural materials with superior conductive properties. The bonds typically formed during the transition from sol to gel are based on the condensation reactions between metal atoms, which are mediated through oxygen atoms. These attributes render the sol-gel method highly effective for synthesizing metal oxide/ CNT composites with optimized performance characteristics.

The sol-gel method has demonstrated significant benefits in the synthesis of hydrous RuO_2_/graphene sheet composites for use in high-performance electrochemical capacitors. This technique provides a robust framework for developing graphene-based composites with notably enhanced electrochemical performance. [44]. Recently, anisotropic ZnO/rGO and LiFePO_4_/C nanocomposites have been recognized as high-quality electrode materials for SCs and batteries, respectively. [45, 46]. Furthermore, the sol-gel process has been effectively applied to encapsulate silicon carbide (SiC) particles with magnesium oxide (MgO) and magnesium aluminate (MgAl_2_O_4_) spinel. This research highlights the advanced electrochemical properties of these composites, showcasing their potential applications in SCs, fuel cells, and electronic devices, thereby affirming the versatility and efficacy of the sol-gel method. [47].

#### Hydrothermal and solvothermal synthesis

**Fig. 3:**
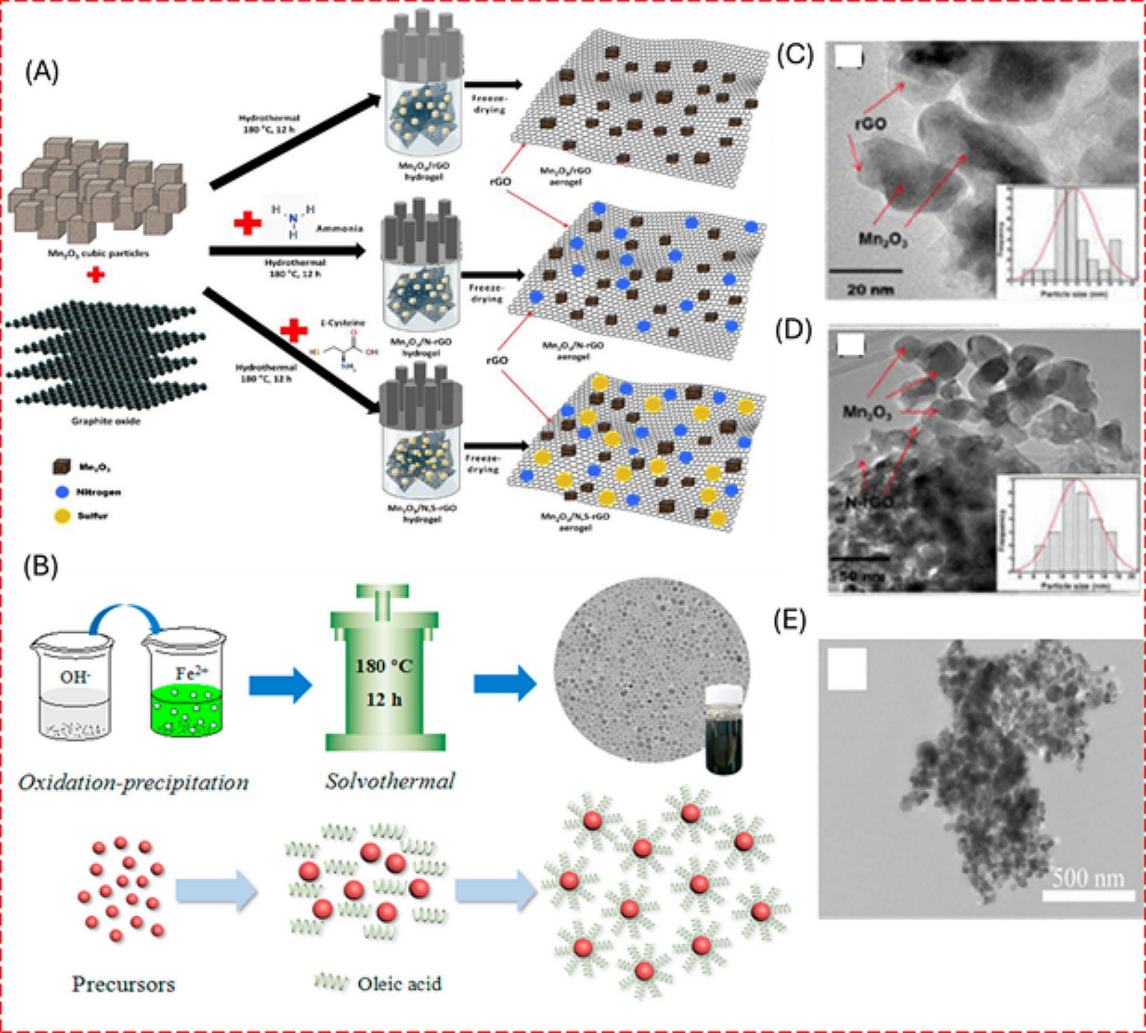
Schematic diagram representation of synthesis procedures involved in (**A**) hydrothermal and (**B**) solvothermal synthesis. (**C**, **D**) TEM images of Mn_2_O_3_/rGO aerogel at 20 nm and 50 nm respectively and (**E**) Fe_3_O_4_ NPs. [50, 51]

In hydrothermal and solvothermal synthesis, a mixture of solvents and precursors is sealed within a pressure-resistant vessel. The type of solvent chosen significantly impacts the synthesis process, as it acts as both a medium and a catalyst, facilitating the growth of 1D or 2D nanomaterials through heterogeneous reactions. Hydrothermal synthesis is characterized using aqueous solutions, whereas solvothermal synthesis utilizes organic solvents or other non-aqueous liquids. Both methods require the metal precursors to react under controlled high-temperature and high-pressure conditions to form nanomaterials [48]. Common solvents for the latter include alcohols, glycols, and supercritical fluids such as CO_2_ [49]. Figure 3 describes the hydrothermal and solvothermal processes involved in the preparation of the Mn_2_O_3_/rGO and Fe_3_O_4_ nanoparticles [50, 51]. Microwave-assisted hydrothermal synthesis has further optimized these processes. By directly targeting materials with microwave energy, this approach minimizes energy losses and boosts synthesis efficiency. It also accelerates reaction rates, facilitating rapid nucleation and growth of nanomaterials essential for crafting materials with defined nanostructures and properties [52].

Hydrothermal and solvothermal synthesis methods are exceptionally effective for fabricating precisely engineered zeolites and nanostructures with designated lattice structures and chemical compositions, making them ideal for applications such as highly efficient SCs. These methods allow meticulous control over parameters such as temperature, pressure, pH, and reaction duration, enabling fine-tuning of particle size, shape, and crystallinity [53]. As a result, a diverse range of morphologies, including nanosheets, nanoflakes, and nanoparticles, can be produced. This precise control is crucial for developing materials tailored for specific functionalities. Additionally, by adjusting these conditions, it is possible to create integrated and composite nano-configurations, offering a broader spectrum of structural configurations than other synthesis methods like sol-gel or exfoliation [54]. This advantage underscores the superior capability of hydrothermal and solvothermal methods in advanced material engineering.

Considering the vast array of materials synthesized using hydrothermal and solvothermal methods, an exhaustive presentation of individual examples would not effectively capture the full scope of advancements made in this study. Detailed information on specific nanomaterials and nanocomposites synthesized via these techniques is systematically cataloged in Tables 1 and 2. These methods are especially notable for their capacity to engineer a diverse array of compositions and structural configurations. For instance, we have developed unique composite structures such as a double sandwich-like Co_2_SiO_4_/derivatives with GO composite [55], demonstrating flexibility in layering and material integration. Additionally, various nano configurations of MnCO_2_O_4_, ranging from nanorods and porous/nonporous nanospheres to nanoflakes and nanosheets, illustrate the ability to precisely control nanostructure morphology.

**Table 1:** Metal oxides and its composites synthesis method, morphology and specific capacitance/capacity comparison for SCs

S. No	Material	Method	Capacitance / Capacity	Ref.
1.	MnHCF-MnO*x*/ErGO	Coprecipitation method	467 F g^− 1^ at 1 A g^− 1^	[114]
2.	G/AME-MoO_2_	Fused deposition modelling	1212 F g^− 1^ at 1.48 A g^− 1^	[155]
3.	Graphene/VO_x_	Facile laser-scribing process	1110 F g^− 1^ at 20 mVs^− 1^	[115]
4.	CoMn_2_O_4_/C hollow spheres	Hydrothermal reaction and subsequent calcination	216 F g^–1^ at 0.1 A g^–1^	[109]
5.	(Co_3_O_4_ − PBMCO)	Pechini synthesis method and calcination	1571 F g^− 1^ at 1 A g^− 1^	[156]
6.	O_v_-Cu-Co_3_O_4_@C	Hydrothermal followed by calcination	180.2 F g^− 1^ at 1 A g^− 1^	[107]
7.	MnO_2_–CNTs	Probe ultrasonication	115.1 F g^− 1^ at 1 mV s^− 1^	[29]
8.	CNT/High Mass Loading MnO_2_/ Graphene	Graft-deposit-coat strategy	3.38 F cm^− 2^ at 1 mA cm^− 2^	[100]
9.	K_2_Ti_6_O_13_ Nanoparticle‑Loaded Porous rGO	Aerosol spray pyrolysis and post-heat treatment	275 F g^− 1^ at 0.5 A g^− 1^	[157]
10.	MXene/MoO_3_	Hydrothermal and vacuum filtration method	545 F g^− 1^ at 3 mV s^− 1^	[30]
11.	S-doped TiO_2_/ C nanofibers	Electrospinning method	114 mAh g^− 1^ at 5000 mA g^− 1^	[101]
12.	CNT@MnO_2_	Modified one-pot synthesis method	2.91 F cm^− 2^ at 20 mA cm^− 2^	[158]
13.	N-STC/Fe_2_O_3_ nanocomposite	Polymer mixture and carbonization	267 F g^− 1^ at 2 mV s^− 1^	[116]
14.	NiFe_2_O_4_ QD/G	Biomimetic mineralization synthetic strategy	697.5 F g^− 1^ at 1 A g^− 1^	[112]
15.	GO- EDA-BisFc/PANI composite	interfacial polymerization	272 mAh g^− 1^ at 2.5 A g^− 1^	[159]
16.	C@Co, CoO/Co_2_SiO_4_/ rGO	Hydrothermal and carbonization	687 mF⋅cm^− 2^ at 1 mA cm^− 2^	[55]
17.	Ppy-NCS@Ni@SiNWs	Hydrothermal	102.83 F g^− 1^ at 0.5 Ag^− 1^	[160]
18.	Co-MoS_2_@Cu_2_MoS_4_	Hydrothermal	150 F g^− 1^ at 1 A g^− 1^	[56]
19.	(GO) (CCS@GO) hybrid electrode	Hydrothermal	192.8 Fg^− 1^ at 1 Ag^− 1^	[161]
20.	Co(OH)_2_/Ni(OH)_2_	Solvothermal	1.4 F cm^− 2^ at 2 mA cm^− 2^	[162]
21.	Vanadium oxide@molybdenophosphate	Electrodeposition	3,954 mF cm^− 2^	[163]
22.	PANI@CNT-BC	in-situ polymerization	235.7 mF cm^− 2^ 0.5 mA cm^− 2^	[164]
23.	polyurethane/porous wood	Chemical polymerization	157.7 F g^− 1^ at 0.3 A g^− 1^,	[165]
24.	Zn-Co-OH/MnO_2_	Hydrothermal	1247.1 C g^− 1^ at 1 A/g	[166]
25.	Ethyl viologen-functionalized reduced graphene oxide	hydrothermal	222.7 F g^− 1 ^ at a 0.5 A g^− 1^	[167]
26.	CoS2@gC/rGO nanocomposite	hydrothermal	233 F/g at a 1.5 A g^-1^	[168]
27.	Fe_2_N@Fe_3_O_4_ core-shell	microwave	34 F g- 1 at 0.5 A g^-1^	[169]
28.	MXene/TiO_2_-graphene	sonicating	35.5 F/g at 1 A g^-1^	[58]
29.	AGC@MnO_2_	carbonization	1260 F g^− 1^ at 1 A g^-1^	[170]
30.	poly (Indole-6-carboxylic acid) adorned with nanorod MnO2	hydrothermal	43.01 mF cm^− 1^	[57]
31.	TiO_2_ NFs@Au@MnO_2_	Electrodeposited hydrothermal	223.75 F g^− 1^ at a 0.5 A g^− 1^	[171]
32.	NiAl LDH@Mn_3_O_4_@Co-MOF	Electrodeposited hydrothermal	151.76 F g-1 at 1 A g^-^^1^	[59]
33.	rGO@Mn_2_V_2_O_7_	hydrothermal	112 F g^− 1^ at 10 A/g	[172]
34.	Co_3_O_4_@NiO nanosheet arrays	Chemical bath deposition	715 F g^− 1^ at 0.5 A g^− 1^	[173]
35.	FeCo_2_O_4_ nanosheets	solvothermal	853.8 F g^− 1^ at 5 A g^− 1^	[174]
36.	CuCo_2_O_4_ nanosheets	electrodeposition	1473 F g^− 1^ at 1 A g^− 1^	[175]
37.	V_2_O_5_ nanosheets	solvothermal	253 F g^-^^1^ at 1 A g^− 1^	[176]
38.	MnO_2_ nanosheets	Soft template	774 F g^− 1^ at 0.1 A g^− 1^	[177]
39.	Pd doped NiCo_2_O_4_ nickel foam	Hydrothermal	2484 F g^− 1^ at 2 A g^− 1^	[178]
40.	2D-Fe_2_O_3_ nanoplates	Hydrothermal	347 F g^− 1^ at 1 A g^− 1^	[179]
41.	2D-VO_2_ nanosheet	Hydrothermal	405 F g^− 1^ at 2 A g^− 1^	[27]
42.	2D-Co_3_O_4_ nanosheets	Hydrothermal	1500 F g^− 1^ at 1 A g^− 1^	[180]
43.	Plate like MnO_2_/carbon nanosheets	Low temperature oxidation method	339 F g^− 1^ at 0.5 A g^− 1^	[181]
44.	2D-SnO_2_ nanoplates	hydrothermal	210 F g^− 1^ at 2 A g^− 1^	[182]
45.	NaMnO nanosheets	molten salt	131 F g^− 1^ at 1 A g^− 1^	
46.	2D CuO nanosheets	hydrothermal	1057 F g^− 1^ at 2 A g^− 1^	[183]
47.	2D-MnO_2_ nanosheets	Ball milling	306 F g^− 1^ at 0.2 A g^− 1^	[28]

**Table 2:** Metal oxides and its composites synthesis method, morphology and specific capacity comparison for battery

S. No.	Material	Synthesis method	Morphology	Specific capacity (mAh g^− 1^)	Reference
1	Fe_3_O_4_	Thermal decomposition	Cube shape	803	[184]
2	Fe3O4@C	Hydrothermal	Porous tube	800	[185]
3	Co3O4	Solvothermal	Peanut shape	700	[186]
4	h-Co3O4@RGO	Pyrolysis method	Porous spherical	1154.2	[187]
5	Cu/TiO_2_	Freeze-drying	Porous layered structure	223	[188]
6	Co_3_O_4_/TiO_2_/carbon	Hydrothermal	Rattan-like	1239	[189]
7	ZnO/C	Sol-gel method	Capsule-like	790	[190]
8	MnO	Hydrothermal method	Six-branched star-like	834.4	[191]
9	MnO@C	Hydrothermal method	Hollow sea urchin-like	839	[192]
10	MoO_3_-NiO @C	Two step heat treatment	Coral globular-like	944	[193]
11	SnO_2_@G	Hydrothermal	Porous layered	406	[126]
12	Co_3_O_4_/NC	Hydrothermal	Nanowire	1115.7	[194]
13	Co_3_O_4_@Graphene	Hydrothermal	Honeycomb shape	1015	[195]
14	Co_3_O_4_/MXene	Hydrothermal	Sandwich	1005	[196]
15	Si@TiO_2_@rGO	Sol-gel	Sandwich	1135.1	[197]
*16*	*N*-doped Ti_3_C_2_@TiO_2_	Hydrothermal	Sandwich	302	[198]
17	NiO/Graphene	Hydrothermal	Flower-like	700	[199]
18	ZnO/Co_3_O_4_-NC	Template-assisted	Spherical	750	[200]
19	TiO_2_@C@ZnO	Atomic deposition & hydrothermal treatment	Nanorod	1154	[201]
20	ZnO@G	Ball milling	Block structure	720	[202]
21	CoO/ZnO-NrGO	Solvothermal	Three-dimensional layered	600	[203]
*22*	*N*-MnO/rGO	Hydrothermal	Cocoon pupa	1020	[204]
23	MnO-graphene	Hydrothermal	Nanowire	1185	[205]
24	ZnO/ZnS@NC/CNTs	Molecular-assisted	Hollow tube-like	1020.6	[206]
25	MnO/C@rGO	Solvothermal method	Diamond shape	1536.4	[207]
26	Fe_3_O_4_@PrGO	Redox deposition	Three-dimensional mesh	2136	[208]
27	ZnO@C	Carbonization	Nanosheet-like	990	[209]
28	C/Fe_3_O_4_/rGO	Solvothermal method	Sandwich-like	844	[210]
29	ZnO/H-rGO	In-situ etching method	Honeycomb shape	942	[211]
30	MnO/Mn_3_O_4_/NG	Laser-inducing	Spongy	699	[212]
31	NiO@Co_3_O_4_@GQDs	Solution heat treatment	Multi-layer hollow sphere	1158	[213]
32	NiO/NC	Hydrothermal and calcination	Fluffy ball-like	450	[214]
33	MnO@SNC	Pyrolysis	Nanorod-like	955.5	[215]
34	SnO2/rGO	Hydrothermal	Crystal like	800	[216]
35	CuO/CNF	Electrospinning & carbonization	Nanofibers	528	[217]
36	Fe_2_O_3_/N-graphene	Hydrothermal	Nanosheet	438	[218]
37	SnO_2_/Fe_3_O_4_	Galvanic replacement	Quantum dots	1300	[219]
38	C/Fe_3_O_4_	Ultrasonic spray pyrolysis	Quantum dots	460	[220]
39	Fe_3_O_4_@NCm	In-situ polymerization	nanorods	760	[221]
40	H-TiO_2_/C/Fe_3_O_4_@rGO	Ultrasonic and vacuum filtration	Core shell	867	[222]
41	SiO_x_@Fe_3_O_4_@FLG	Ball milling	Egg-like	833.4	[223]
42	Co_3_O_4_/TiO_2_	Precipitation & Heat treatment	Hollow polyhedron	642	[224]
43	CuO/Cu_2_O@CeO_2_	Pyrolysis	Porous	473	[225]
44	NiFe_2_O_4_/Fe_2_O_3_	Annealing	Nanotubes	423.6	[226]
45	NiFe_2_O_4_@TiO_2_	Hydrothermal	Nanorods	1034	[227]
46	CuCo_2_O_4_@C	Calcination	Concave polyhedron	740	[228]
47	Li_4_Ti_5_O_12_/C	Thermal annealing	Tablet-like	277	[229]
48	ZnFe_2_O_4_/C@NCNT	Pyrolysis	Nanotubes	11,000	[230]
49	Fe_2_O_3_@NiCo_2_O_4_	Thermal annealing	Porous Nanocages	1311.4	[231]
50	MnO/C	Hydrodynamic induction	Hollow tube-like	845	[232]

In transition metal dichalcogenides (TMDCs), materials such as Co-MoS_2_/Cu_2_MoS_4_ [56] highlight the synthesis of complex hybrid materials that integrate multiple functionalities for enhanced electrochemical performance. The synthesis of Poly(indole-6-carboxy acid) nanowire composites with β-MnO_2_ nanorods (6-PICA/β-MnO_2_–NRs) [57] showcases the integration of organic and inorganic phases to achieve synergistic properties. Moreover, the ferric ions-assisted self-assembly of MXene/TiO_2_-graphene aerogel composites (MXene/TiO_2_-Fe-G) [58] represents a sophisticated example of utilizing metal ion coordination to guide the assembly of highly porous, three-dimensional structures with high surface areas. Lastly, the NiAl LDH/Mn_3_O_4_/Co-MOF ternary composites [59] demonstrate the potential for creating layered hybrid materials that combine multiple metal oxides and organic frameworks for targeted applications. These presented examples underscore the technical sophistication and innovative potential of hydrothermal and solvothermal synthesis methods in creating precisely tailored materials for advanced applications across various technological domains.

#### Chemical vapor deposition

Chemical Vapor Deposition (CVD) is a vital technique for fabricating high-quality materials through the chemical reactions of volatile precursors. Recognized as one of the most effective bottom-up synthesis methods, CVD is especially acclaimed for producing commercial-grade graphene and GO [60]. It is extensively used to synthesize a variety of thin films and nanostructures under rigorously controlled temperature, pressure, and chemical environment conditions. This precise control ensures uniform deposition on templates, resulting in high reproducibility of the materials produced. The process involves the decomposition or reaction of gas-phase precursors that, when interacting with a heated substrate, produce a solid deposit while typically gaseous by-products are evacuated.

Due to this mechanism, high-temperature processing is an essential part of CVD. However, the integration of microwave plasma in CVD processes enables the synthesis of materials at lower temperatures compared to their traditional counterpart. This lower-temperature processing can be crucial for protecting temperature-sensitive substrates and minimizing thermal stresses within the material structure. Furthermore, plasma plays a pivotal role in precisely controlling the nanostructure and related qualities of materials. For instance, In_2_O_3_ nano blades synthesized via plasma-enhanced CVD (PE-CVD) show enhanced electrochemical properties as anode materials. They achieved a reversible capacity of 580 mAh/g after 100 cycles, marking a substantial improvement over thin films produced by conventional methods [61].

**Fig. 4:**
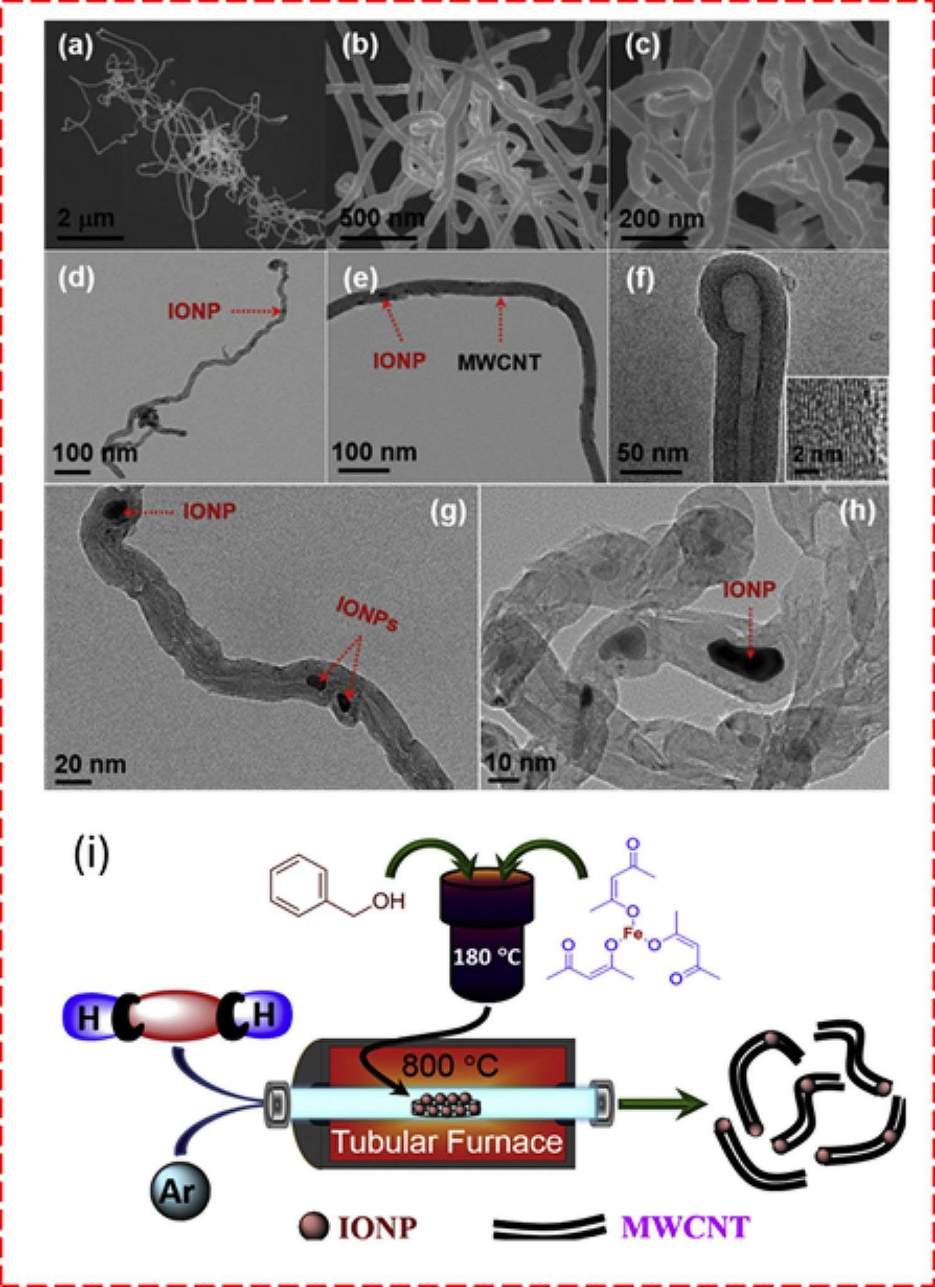
(**a**-**c**) FESEM images with different magnification, (**d**–**f**) TEM images, (**g**) and (**h**) HRTEM images of synthesized iron -oxide nanoparticles- multiwalled carbon nanitubes IONP-MWCNTs hybrid. Inset of (**f**) HRTEM image of the walls of MWCNTs within the synthesized IONP-MWCNTs hybrid. (**i**) Schematic diagram representation of synthesis procedures of CVD methods for IONP-MWCNTs hybrid [63]

Note that the primary advantages of chemical vapor deposition (CVD) include its ability to produce uniform, high-purity materials with customizable structural properties. This technique is particularly valuable for applications that require thin films with excellent conformity or multi-layered structures. Indeed, the multilayered hematite (Fe_2_O_3_) and rGO, synthesized through CVD, have demonstrated exceptional specific capacity and charge/discharge cycling stability, attributable to their improved structural integrity [62]. Electrospun carbon nanofibers with Fe/Fe_3_O_4_-encapsulated carbon nanotubes (CNTs) (Fig. 4) [63] and carbon nanocoils on nickel foam, fabricated using CVD techniques for SC electrodes, have been also reported [64].

#### Green synthesis

Green synthesis methods focus on environmentally friendly and sustainable approaches to produce metal oxide composites [65]. The main aim is to reduce or eliminate the use of hazardous chemicals, minimize energy consumption, and utilize renewable resources. Metal oxide composites can be synthesized via plant extracts and microbial synthesis methods that can be used as reducing agents, and stabilizing agents to metal oxide nanoparticles respectively [66]. Incorporating renewable biomaterials such as cellulose, chitosan, and other natural polymers as templates or supports in the synthesis of metal oxide composites [67]. These materials are biodegradable and contribute to the green credentials of the synthesis process. In this regard, the choice of solvent can significantly impact the environmental impact of the synthesis process [68]. The summary of the preparation methodologies along with the advantages and the limitations are provided in Table 3.

**Table 3:** Summary of the advantages and disadvantages of the preparation methods

Top-down approaches		
Synthesis method	Advantages	Limitations
Exfoliation	- Ideal for creating two-dimensional materials like graphene. - Allows exploration of quantum properties in materials. - Suitable for producing a variety of metal oxide nanosheets.	- Often results in low yield, which limits large-scale production. - Produces small flake sizes that may not be ideal for all applications. - Risk of contamination during the process, affecting material purity.
Ball milling	- Enables precise control over particle size and distribution. - Useful for a wide range of materials. - Scalable for industrial production [146].	-Mechanical forces can induce unintended structural changes. -Contamination from milling media is a concern. - Energy-intensive process. - Heat and noise pollution [147].
Lithography	- Extremely precise, allowing for complex nanostructure fabrication. -Essential for advanced electronics and nanodevice prototyping. - Precise alignment in multilayer fabrication process [148].	- Requires sophisticated and expensive equipment. [36] - Process complexity leads to higher operational costs. - Limited to surface patterning, not bulk material synthesis.
**Bottom-up approaches**		
**Synthesis method**	**Advantage**	**Limitations**
Sol-gel	- Low-temperature processing protects temperature-sensitive materials. [147] - Can produce high-purity materials with controlled properties. - Versatile for coating and embedding various substrates	- Prone to shrinkage and cracking during drying and firing. - Lengthy processing times hinder quick production cycles. - Scaling up remains challenging due to process sensitivities. - limited precursors [149].
Hydro- and Solvo-thermal	- Allows meticulous control over particle size, shape, and crystallinity - Can produce a diverse range of morphologies. - Effective for synthesizing complex metal oxides and composites.	- High-pressure and temperature conditions limit the types of materials that can be used. - Energy-intensive, impacting operational costs. - Requires specific equipment and safety measures. - Slow process [143]
Chemical vapor deposition	- Produces uniform, high-purity materials with customizable properties. - Suitable for thin film and advanced material production. - Capable of producing materials with excellent conformity and multi-layered structures. [149].	- Requires controlled environments and high temperatures. - Complex setup and maintenance. - High operational costs due to energy and precursor material requirements. - Safety risks while using hazardous precursors [150].
Microwave assisted method	- Highly efficient method for nanoparticle synthesis. - Straightforward, rapid volumetric uniform heating leading to a significant increase in reaction rate. [151]	- Not all materials are compatible for microwave irradiation [152].
Green synthesis	-Utilizes non-toxic, renewable resources, reducing environmental impact. -Supports the synthesis of biodegradable and sustainable materials. - Often lower in cost due to the use of natural precursors [153].	- Typically offers less control over the material’s final properties compared to other methods. - Material properties may vary due to natural variability in biological agents. - Scale-up can be challenging without compromising green principles - Purity of synthesized material is low and contaminants issue [154].

## Materials perspectives in energy storage system

Carbon and its various forms, such as graphene, fullerenes, and CNTs, stand out as primary materials in energy conversion and storage technologies as they offers higher surface area and more electroactive sites as well as functional flexibilities [69, 70]. Recently, MXenes were also introduced as promising materials for the electrodes for energy storage systems because of chemical/structural stabilities and excellent conductivity [71, 72]. The remarkable success of graphene/its derivatives and MXenes in energy storage technologies has led to increased research into other 2D materials and their composites, attributed to their layered structures and the fast ion intercalation/deintercalation capabilities during the charge storage process [73]. This surge in interest aims to leverage these unique properties of materials for enhanced energy storage solutions. This includes a diverse array of low-dimensional nanomaterials and compounds like metal, metal oxides, metal chalcogenides, metal phosphates, metal carbides, nitrides, and jarosites [74]. The development of composites from these materials would facilitate the creation of innovative materials. This enhancement significantly improves performance, selectivity, and efficiency, paving the way for advanced energy storage solutions. From the series of materials available for energy storage, we here focus on utilizing low-dimensional metal oxide and their respective composites.

The use of metal oxide composite structures introduces new perspectives on material innovation [75, 76]. Nanocomposite configurations, which combine distinct unit materials with unique physical and chemical properties, are expected to be a groundbreaking approach due to their ability to selectively tailor synergistic effects. However, the use of metal oxide composites as electrodes presents several challenges [11, 13]. One such issue is the tendency of metal oxide nanoparticles to aggregate, which reduces the effective surface area and impairs electrochemical performance. Some metal oxides may also exhibit instability or reactivity in aqueous environments, limiting their applicability in settings exposed to water. Furthermore, the complete synthesis and fabrication processes of the composite structure can be costly.

### Synergistic effect of using metal oxides

The synergistic effect of metal oxide composites in energy applications, particularly as electrodes of SCs and batteries, is significant and offers several advantages. Metal oxide composites turn out to improve SCs by delivering higher specific capacitance and energy density alongside a rapid charging/discharging rate. Metal oxide composites with tailored morphologies and structures offer increased surface area, facilitating better ion adsorption and desorption kinetics [77]. Composite structures of metal oxides and carbonaceous materials can prevent the restacking of layers, ensuring efficient utilization of active materials and prolonged cycle life [78]. The development of diverse sustainable synthesis techniques for metal oxides is crucial, taking into account their morphological, compositional, and supercapacitive characteristics. This encompasses the exploration of SC designs and configurations, the classification of SCs according to electrode materials, and an in-depth examination of metal oxide-based pseudocapacitive and battery-type electrodes.

In batteries, the use of metal oxide composites can improve electrode stability and prevent electrode degradation during charge/discharge cycles. Composite electrodes with metal oxides exhibit improved charge storage capacity and cycle stability, resulting in a longer battery lifespan and better performance [79]. The synergistic effect between metal oxides and other materials allows for the optimization of electrode-electrolyte interfaces, minimizing side reactions and enhancing battery efficiency [80].

**Fig. 5:**
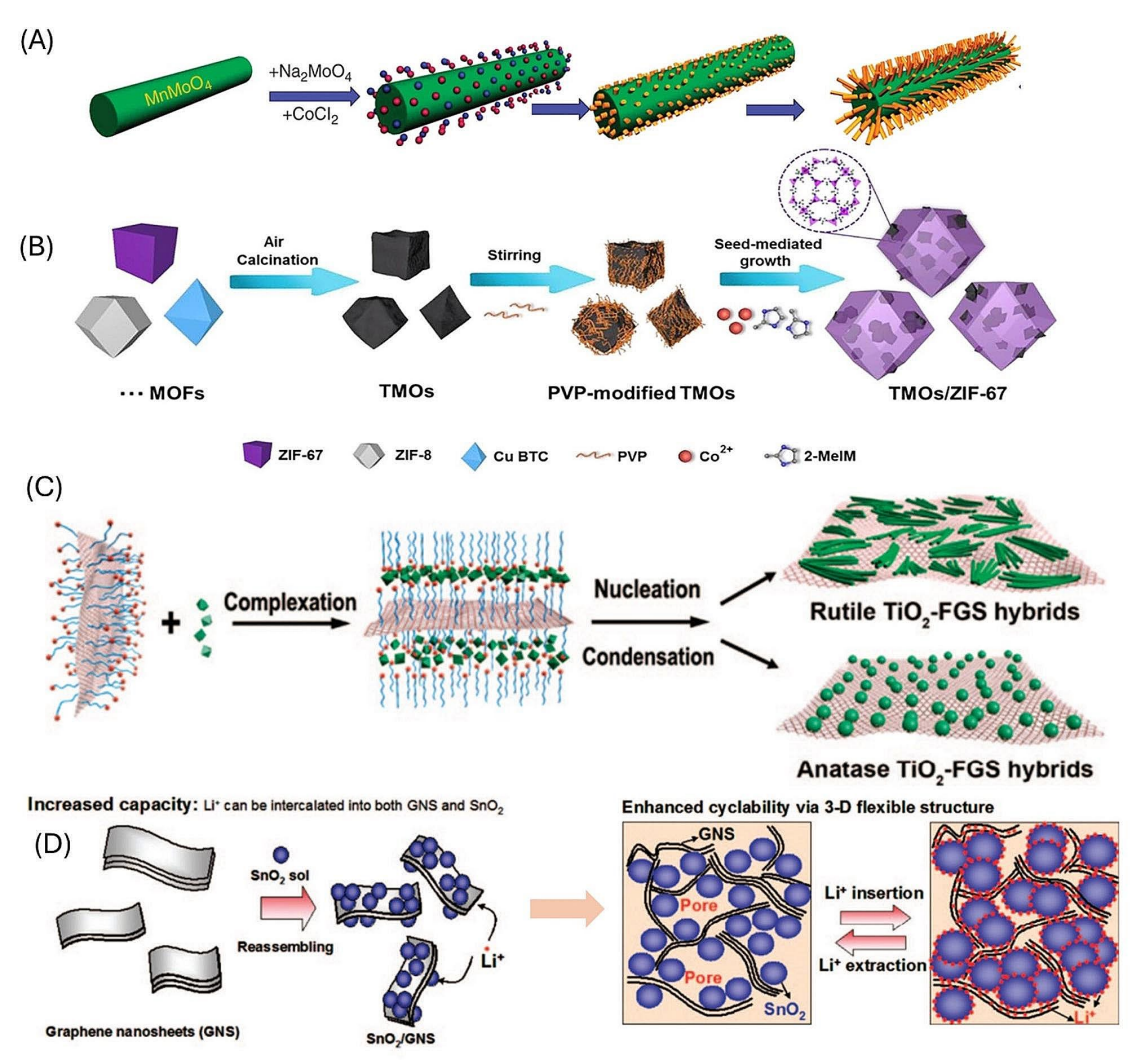
The various structures of the metal composites grown with different architecture such as (**A**) MnMoO_4_ nanowires in CoMoO_4_ nanorods, (**B**) MOF @ TMO composite with seed-mediated growth, (**C**) TiO_2_- Graphene sheet composites and (**D**) SnO_2_ decorated on graphene nanosheets respectively [141–144]

**Fig. 6:**
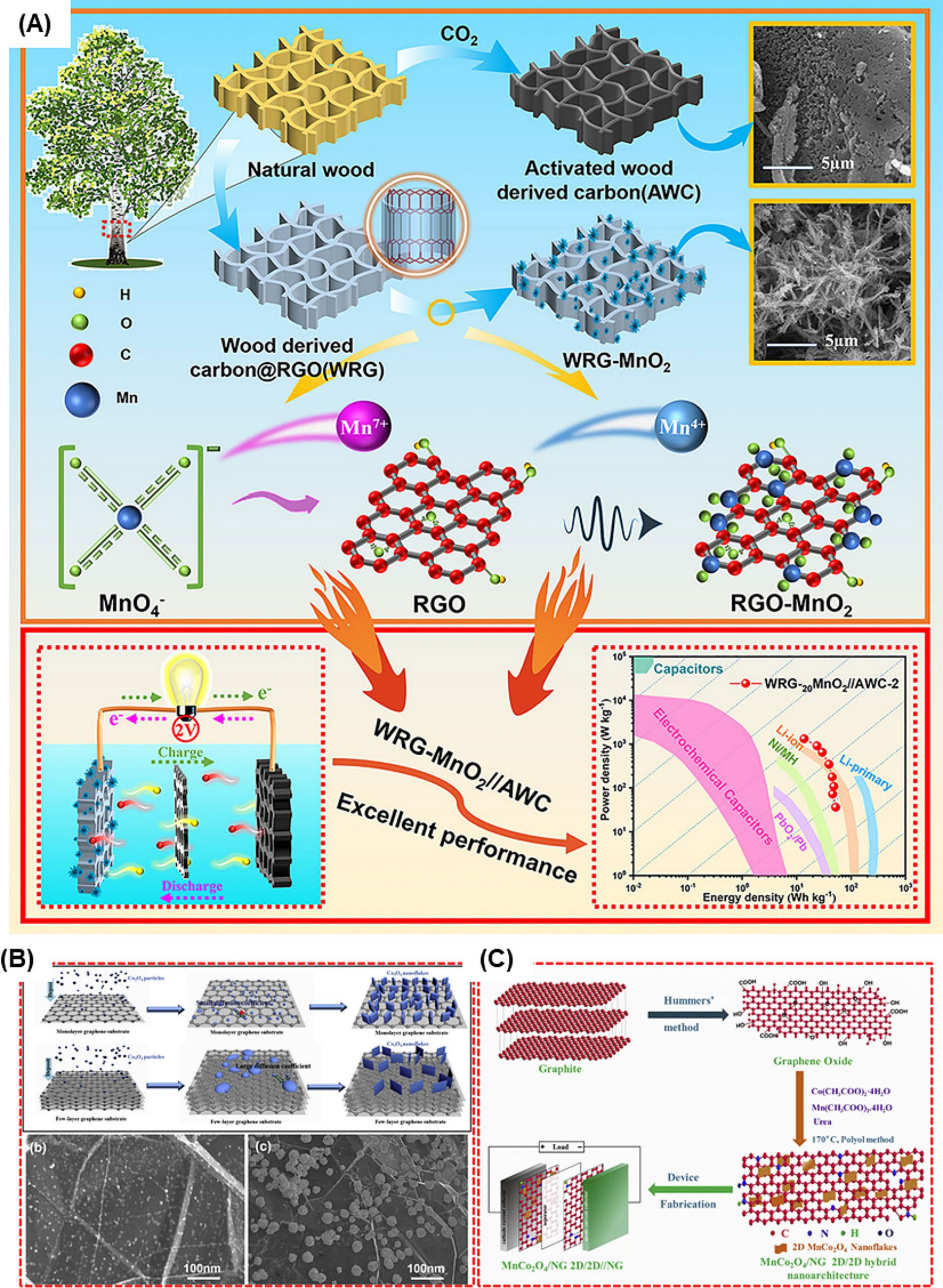
(**A**) Schematic representation of the preparation for composite with the synergetic effect of EDL and pseudocapacitive material. (**B**) Pictorial representation of Co_3_O_4_ nanoflakes/graphene composite synthesize in different conditions. (**C**) Schematic illustration for the preparation of MnCo_2_O_4_/NG 2D/2D hybrid nanoarchitectures for ASC [84, 87, 88]

Note that the efficiency may be directly affected by several factors such as crystallinity, specific surface morphology, and electronic conductivity of the metal oxides. Metal oxides store charge via faradaic redox mechanism mostly with the change in phase during the charging process whereas some of the metal oxides such as MnO_2_ and RuO_2_ follows the pseudocapacitive nature of storage [81]. Crystallinity significantly influences the charge storage capabilities of metal oxides; the amorphous phase often outperforms the crystalline phase due to better ion diffusion channels enhancing electrochemical performance. Additionally, the morphology of metal oxides, such as nanosheets and nanoflakes, affects their properties, offering superior physical and chemical attributes compared to nanoparticles and spherical structures [82]. A high specific surface area also plays a crucial role, improving electrode/electrolyte interactions and increasing electroactive sites for redox and intercalation reactions [83]. Thus, optimizing the properties of metal oxides is essential for their effective use in electrochemical applications. Various structures of the metal oxide composites are shown in Fig. 5, which resembles the different morphological integration of the electrode materials. For instance, the composite of MnO_2_ with wood derived carbon and rGO shows the synergistic effect in the charge storage such as the pseudocapacitance nature of the MnO_2_ and the double layer capacitance of the rGO produced an excellent electrochemical performance (Fig. 6A) [84].

### Metal oxide composites

#### Metal oxide/graphene composites

Metal oxide/graphene composites hold significant promise for SC applications due to several key factors. The composite structure promotes high conductivity by facilitating electron transfer from the graphene layer to the metal oxides. This boosts hole concentration in graphene, improving overall conductivity. Metal oxides are often sandwiched between graphene nanosheets, preventing restacking of graphene layers and ensuring efficient utilization of the materials. In MnO₂/graphene composites synthesized via electrostatic co-precipitation, which entails mixing oppositely charged components in a solvent to promote aggregation and precipitation through electrostatic interactions, an enhanced electrochemical performance exceeding that of pristine MnO₂ and graphene alone has been observed [85]. This composite leverage exceptional electrical conductivity of graphene for rapid charging and discharging, alongside substantial capacitance of MnO₂ to augment energy storage capacity. Such a synergistic integration not only amplifies energy and power density but also significantly enhances cyclic stability. Consequently, this composite structure outperforms the individual capabilities of either material, marking a notable advancement in energy storage technology.

Similarly, the composite structure of flower-like NiO supported on graphene sheets surpasses the performance of either NiO or graphene alone in SCs [86]. The synthesis of NiO on graphene sheets involves a two-step process: initially, graphene is produced by chemically reducing graphite oxide, followed by a hydrothermal treatment of a mixture containing graphene, nickel nitrate, urea, and a surfactant to form NiO precursors. This mixture is then calcined under nitrogen to convert the precursors into nickel oxide. Notably, this composite exhibits a specific capacitance of 346 F/g at 1.5 A/g, significantly exceeding that of bare NiO (220 F/g). The superior performance is attributed to the unique flower-like NiO morphology that increases surface area for electrolyte contact and the conductive graphene sheets that provide structural stability, enhancing the electrochemical performance [86].

Additionally, other studies have explored various composite structures, such as ultrathin MnO_2_/graphene nanosheets and Co_3_O_4_ nanoflake/graphene composites, demonstrating their potential for flexible planar SCs and high specific capacitance (Fig. 6(B)) [87]. These composites were synthesized using a combination of CVD and hydrothermal methods, effectively integrating the unique properties of each component. Further research by Shrestha et al. focusing on MnCo_2_O_4_/nitrogen-doped graphene composites highlights a unique interaction that prevents restacking, significantly boosting the performance of these composites, as depicted in Fig. 6(C) [88].

#### Metal oxide/carbon nanotube composites

**Fig. 7:**
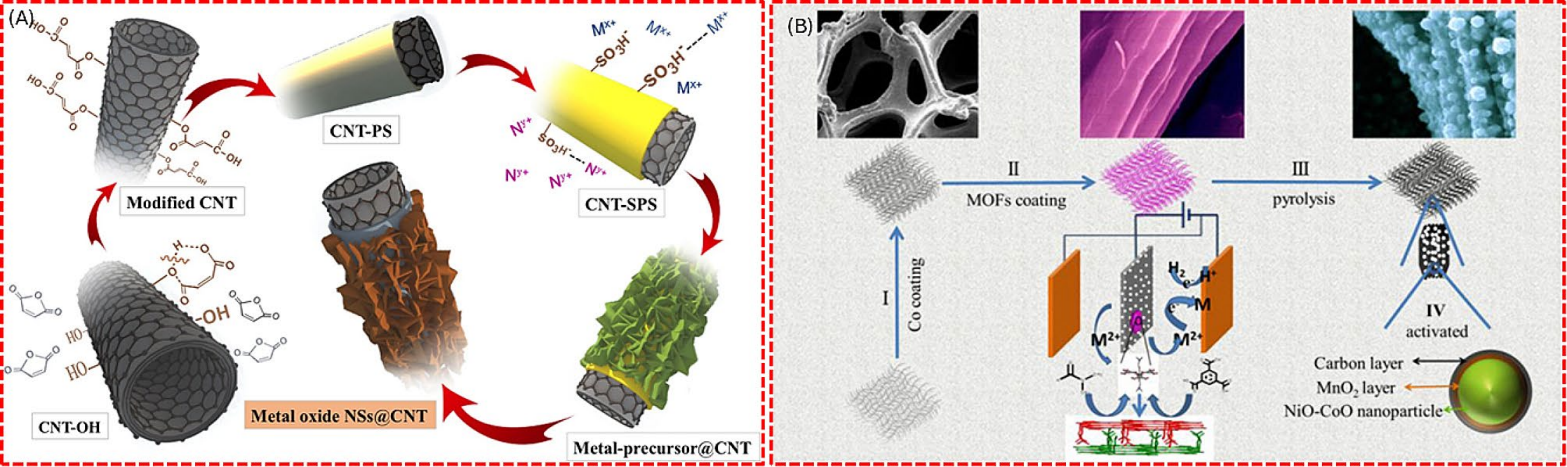
Schematic representation of preparing (**A**) TMO@CNT composites. (**B**) 2D NiO-CoO/MnO_2_/C composite. [90, 92]

CNTs serve as excellent substrates for supporting metal oxides in energy storage applications due to their high conductivity, mechanical strength, and thermal stability [89]. For instance, the transition metal oxide/CNT composites synthesized by wet chemical methods followed by annealing as shown in Fig. 7 (A) demonstrated enhanced electrochemical performance, in which SEM micrographs showed nanosheet structures of NiCoO_2_ on the CNT backbone [90]. The exceptionally high electrical conductivity of CNT enables rapid electron transport within the composite electrode which ensures efficient charge transfer during electrochemical charging and the discharging process.

In another study, Mondal et al. demonstrated the enhanced conductivity and stability of MWCNT/NiCo_2_O_4_ nanosheets, which were fabricated using a microwave-assisted method for use in electrodes [91]. This synthesis involves heating a solution of metal nitrates, urea, and dispersed MWCNTs in a microwave synthesizer at 140 °C for 30 min, facilitating rapid and uniform formation of the precursor materials. The TEM displays the integration of NiCo_2_O_4_ in MWCNT which can offer more electroactive sites and the facilities more space for the ion intercalation/deintercalation during the electrochemical reaction. Similarly, Shadpour et al. explored the effect of CNT functionalization on the properties of metal oxide/CNT composites. It describes the investigation of the various methodologies and strategies of the interaction of CNT and metal oxides along with their impact on the structure, morphology, and performance [43].

#### Metal oxide/activated carbon composites

Activated carbon (AC) is an effective support material for metal oxide/AC composites in SCs, valued for its moderate electronic conductivity and high surface area. Zhang et al. developed a 2D-layered carbon-metal oxide composite electrode through a three-step process that starts with cobalt electrodeposition on Ni foam to form a conductive seed layer as shown in Fig. 7 (B). This is followed by secondary electrodeposition using 1,3,5-benzene tricarboxylic acid to create a Co-metal oxide form, enhancing the electrochemical pathways for ion intercalation. Finally, the assembly is pyrolyzed to embed carbon in the metal oxide, improving conductivity for energy storage applications. This composite demonstrated a high areal specific capacitance, underscoring the potential of metal oxide/AC composites for SC applications [92]. In a different stance, Tugrul et al. reported MnO_2_/AC and NiO/AC composites for SC applications, were synthesized using both hydrothermal and precipitation methods to investigate their effect on the surface chemistry, porous structure, and electrochemical properties on the metal oxide composite-based electrodes. It is proven that the hydrothermal treatment for depositing nano-oxides led to an increase in specific surface area and the presence of oxygen-containing surface functionalities, thereby enhancing the electrochemical properties of the composites. Despite the decrease in specific surface area caused by metal oxide loading, the pseudocapacitive effect of MnO_2_ and NiO, along with the oxygen-containing surface functionalities, contributed to an increase in specific capacitance. Specifically, MnO_2_ and NiO loading resulted in a remarkable 50% and 150% increase in specific capacitance, respectively [93].

#### Metal oxide/polymer composites

**Fig. 8:**
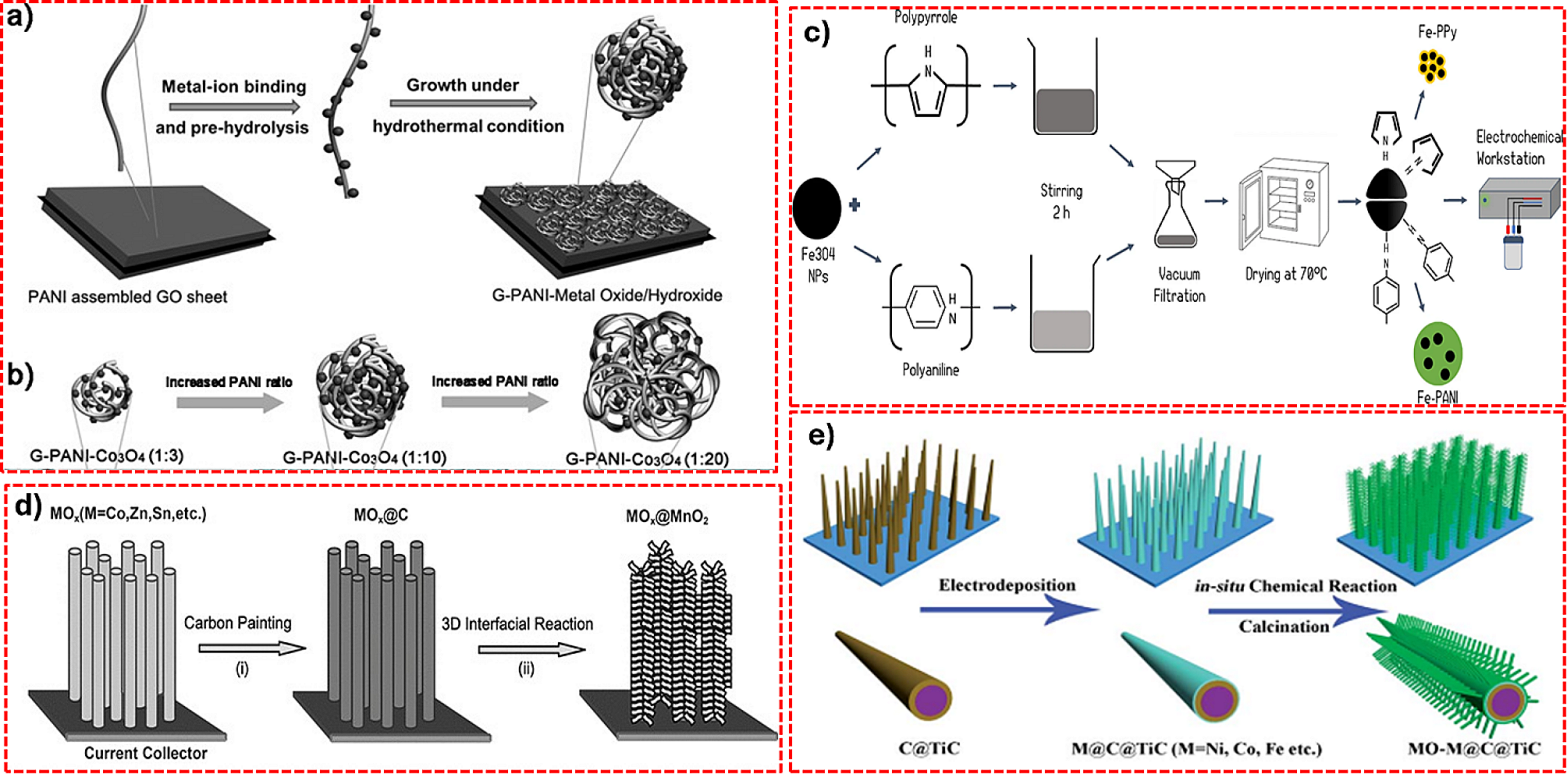
Schematic illustration of the synthesis procedure of (**a**) G-PANI-MOs/MHs composite. (**b**) PANI-Co_3_O_4_ hybrid composite. (**c**) Fe3O4-PPy and Fe3O4-PANI composite. (**d**) Co_3_O_4_ Nanowire@MnO_2_ (**e**) MO–M@C@TiC (M = Ni, Co, Fe, Mn) electrodes. [95, 96, 98, 99]

Conducting polymers, such as polyaniline (PANI), polythiophene (PTH), and polypyrrole (PPy), offer higher conductivity than metal oxides only and are easily integrated into composite structures for SCs [94]. The integration of the conducting polymers in the metal oxide composites produced higher electrochemical properties via improved charge-transport kinetics and flexibility, leading to enhanced energy storage performance with better stability, and rate capability. Li et al. demonstrated controlled growth of 2D ternary hybrids of Co_3_O_4_, PANI, and graphene for SC applications (Fig. 8 (a-b)) [95]. This synthesis involves a process that starts with the functionalization of GO sheets by in-situ polymerization of aniline, which integrates PANI into the GO sheets. After centrifuging, these modified GO sheets are treated hydrothermally with metal salt precursors, allowing the growth of metal oxide or hydroxide nanoparticles directly onto the PANI-modified graphene. In the composite structure with the cobalt oxide, PANI and graphene offer better charge distribution and interaction with the oxide and the carbon-based polymer materials.

By the same token, Jambhale et al. functionalized graphene nanosheets with PANI, followed by the addition of metal salts as precursors and subsequent hydrothermal treatment as shown in Fig. 8 (c). This study investigates the utilization of waste toner-derived Fe_3_O_4_ and its composites with PANI and PPy as electrode materials for SCs. Results showed that the composite synthesized with these conducting polymers achieved the highest specific capacitance of 319 F/g and 286 F/g, respectively, in 4 M KOH electrolyte [96].

#### Metal oxide/metal (oxide) composites

Composite electrodes that combine various metal oxides (metal) can provide superior mechanical stability, enhancing ion and electron transfer pathways and increasing electroactive surface sites. This leads to improved specific capacitance and energy density [97]. For instance, Liu et al. fabricated Co_3_O_4_ nanowire/ MnO_2_ ultrathin nanosheet composites via a hydrothermal method as shown in Fig. 8 (d), achieving superior SC performance [98]. The architecture composites provided efficient charge storage and transfer capabilities with the synergetic effect between the metal oxides, providing the higher electrochemical properties of the materials.

Cheng et al. presented transition metal oxide–metal nanocomposites anchored on conductive nanowire arrays (Fig. 8). The synthesis involves a three-step process for creating metal oxide-metal composites on conductive nanowire arrays. First, transition metals are electroplated onto carbonated titanium nanowire arrays. Next, these metal-coated nanowires are chemically treated with oxalic acid to form metal oxalates directly on the nanowires. Finally, the metal oxalates are transformed into metal oxides through a calcination process, involving heating the materials in air at high temperatures. In particular, they focused on the combinations such as NiO–Ni, Co_3_O_4_–Co, Fe_3_O_4_–Fe, and MnO_2_–Mn, which are integrated with conductive TiC–C core-shell nanowires on a Ti alloy sheet represented in Fig. 8 (e). These composites exhibit a unique 3D nanostructure coupled with high electronic conductivity with the exceptional electrochemical performance of the NiO–Ni/C/TiC nanocomposite. Impressively high capacitance and rate capability are demonstrated, with a specific capacitance reaching 1845 F/g at a charge-discharge current density of 5 A/g. Even after undergoing 500 charge-discharge cycles at an extremely high rate of 100 A/g, the composite maintains a substantial specific capacitance of 811.1 F /g [99].

## Utilizing metal oxide composites in energy storage systems

### Metal oxide composites for supercapacitor

Metal oxide composites are increasingly vital in the development of advanced electrode materials due to their ability to enhance the electrochemical performance of SCs and batteries. These composites improve the internal conductivity of the electrode material and expose many active sites, which are a favor for electrochemical reactions and combinedly to improve the storage performance of the electrode materials.

The flexible electrodes designed for SC applications were crafted using CNT/MnO_2_/graphene coatings on carbon cloth. This optimized nanostructure features excellent conductivity and a large surface area. Specifically, the nanostructured MnO_2_ offers numerous electroactive sites and short ion transport paths, while the CNTs provide conductive channels that enhance electron transport and improve the kinetics of redox reactions. Together, these features enable the electrode to achieve high areal capacitances without sacrificing rate capability, ensuring a broad working voltage and high energy density in asymmetric SCs. Remarkably, this nano-engineered architecture of CNT/MnO_2_/graphene delivers an exceptional areal capacitance of 3.38 F/cm^2^ at a current density of 1 mA/cm^2^ [100].

**Fig. 9:**
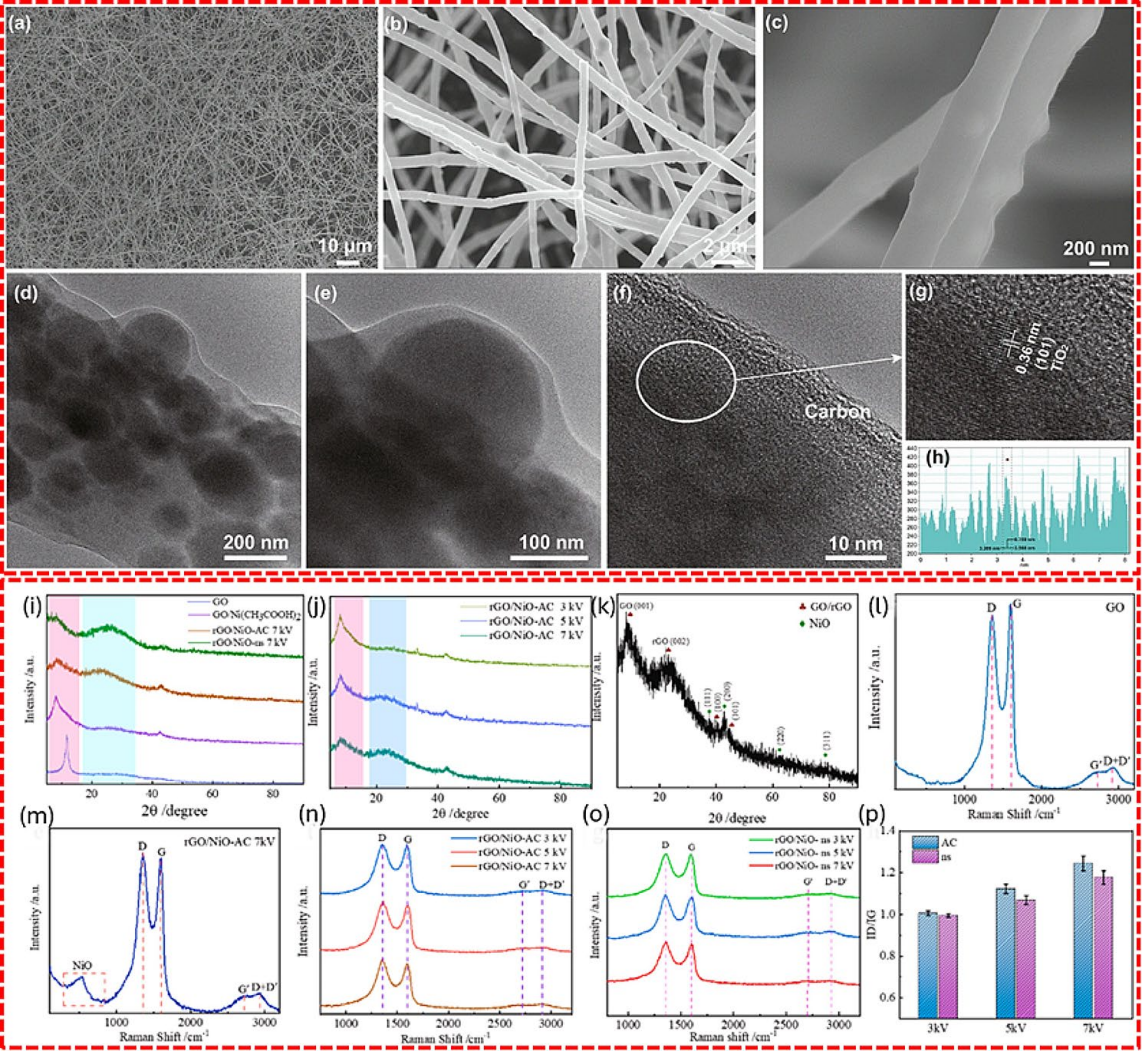
FE-SEM image of electro-spun TiS_2_/S-TiO_2_/C nanofiber (**a**-**c**), Bright field TEM image fiber network (**d**-**e**), The HR-TEM image and lattice fringe spacings of anatase TiO_2_ (**f**-**h**). XRD pattern of NiO/GO nanocomposite and pristine GO under different experimental conditions (**i**-**k**), Raman spectrum of NiO/GO nanocomposite and pristine GO under different experimental conditions (**l**-**o**), I_D_/I_G_ ratio under various experimental conditions (p). [101, 102]

Metal oxide composites such as sulfur-doped TiO_2_ composites combined with TiS_2_ and carbon, prepared using the electrospinning method, and its super capacitive performance was reported [101]. The TiS_2_/S-TiO_2_/C nanofibers feature a uniform, well-dispersed structure within a tightly woven network, where metal oxide and carbon nanoparticles are densely aggregated. These fibers contain nano-scale clusters and display a distinct crystalline structure characteristic of the anatase TiO_2_ (101) plane, as shown in Fig. 9 (a-h). The electrospun nanofibers demonstrate pseudo-capacitance and achieve a specific capacity of 114 mAh/g at a high discharge rate of 5000 mA/g. Clearly, the addition of sulfur significantly enhances the capacity of the TiO_2_/C nanocomposite electrode materials.

The enhanced capacitance of the rGO/NiO composite was also presented, which was fabricated by reducing nickel acetate tetrahydrate and GO using dielectric barrier discharge plasmas. The rGO/NiO composite is characterized by distinctive XRD patterns that differentiate the nanocomposite from pristine GO under various conditions, indicating the crystalline structure and phase purity of the materials. Additionally, Raman spectroscopy reveals the bonding and functional groups within the composite, highlighting its complex molecular interactions and structural integrity, Fig. 9 (i-k). The composite electrode achieved a specific capacitance of 193.3 F/g at 0.5 A/g, indicating improved capacitance of rGO with NiO incorporation [102].

**Fig. 10:**
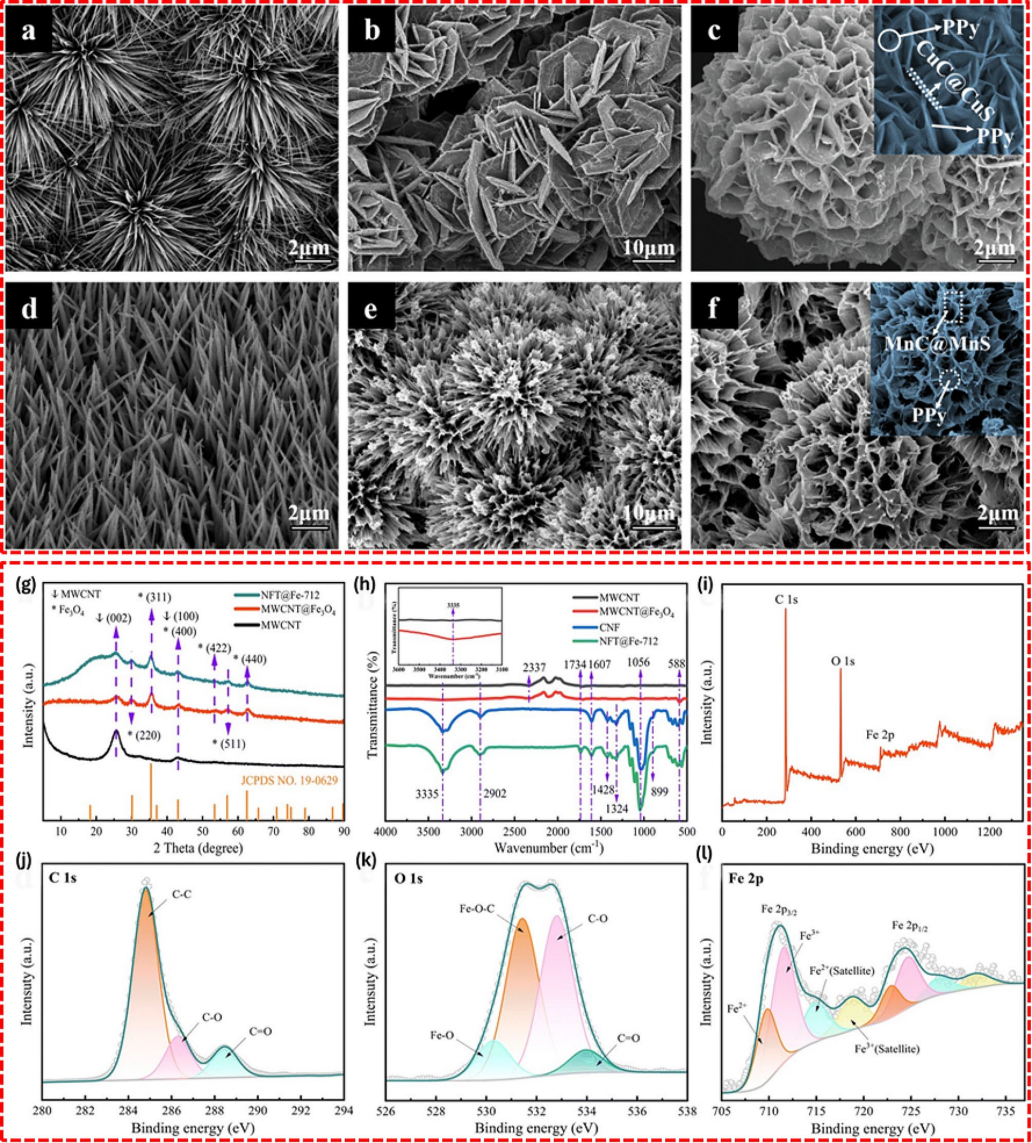
FE-SEM image of the MCo_2_O_4_@MCo_2_S_4_@polypyrrole (M = Cu, Mn) nanocomposite (**a**-**f**). Physicochemical characterization of Fe_3_O_4_/MWCNT/cellulose nanofiber composite (**g**) XRD pattern, (**h**) FTIR, (**i**-**l**) XPS survey spectrum and the core level spectrum of the elements. [103, 104]

Recently, metal oxide-based core-shell composites are getting more attention due to their porous structure, which favors accessing all the active sites of the electrode material by electrolyte ions. The MCo_2_O_4_/MCo_2_S_4_/PPy (M = Cu, Mn) 3D nanoflower core-shell composite is directly grown on nickel foam substrate using a facile hydrothermal method, as shown in Fig. 10 (a-f) [83]. The bimetallic oxides grown on nickel foam almost vertically aligned and covered the entire surface of the substrate. After the pyrole polymerization reaction, a 3D flower-like core-shell structure was formed with the smooth surface of the PPy nanosheets. The prepared nanocomposite exhibits the specific capacitance of 2978.1 F/g for CuCo_2_O_4_/CuCo_2_S_4_/PPy and 4713.4 F g^− 1^ for MnCo_2_O_4_/MnCo_2_S_4_/PPy at the current density of 1 A/g. The capacitance of the MCo_2_O_4_/MCo_2_S_4_/PPy (M = Cu, Mn) nanocomposite reaches its maximum compared to the individual counterparts [103]. A flexible asymmetric SC using Fe_3_O_4_/MWCNT/cellulose nanofiber nano paper through a simple vacuum filtration method for wearable applications. They demonstrated excellent electrical conductivity of 1016.3 S/m without compromising flexibility. The highly conductive, recyclable Fe_3_O_4_/MWCNT/CNF nano paper exhibits extraordinary gravimetric and areal capacitance of 229.9 F/ g and 735.68 mF/ cm^2^, respectively [104].

#### Metal atom doped metal oxide composites for supercapacitor

Metal oxide electrodes typically have low ionic and intrinsic electronic conductivity. By doping or making composites with other good conductive active materials will overcome these issues [105]. Doping involves introducing foreign elements into the metal oxide structure, which can alter the electronic properties and improve conductivity. The benefit of using the composite structure of zinc-doped manganese vanadate (MnZn₃(VO₄)₂) and carbon nanofibers (CNFs) over individual compounds is the enhanced electrochemical performance in SCs. The composite electrode exhibited a specific capacitance of 469 F/g at a current density of 0.5 A/g, which is substantially higher than the specific capacitance values of the pristine electrodes. Additionally, the MnZn₃(VO₄)₂ composite electrode demonstrated exceptional retention, maintaining approximately 82.6% of its initial capacitance even after 5000 charge-discharge cycles. This improvement is attributed to the synergistic effects between the MnZn₃(VO₄)₂ and CNFs [106].

**Fig. 11:**
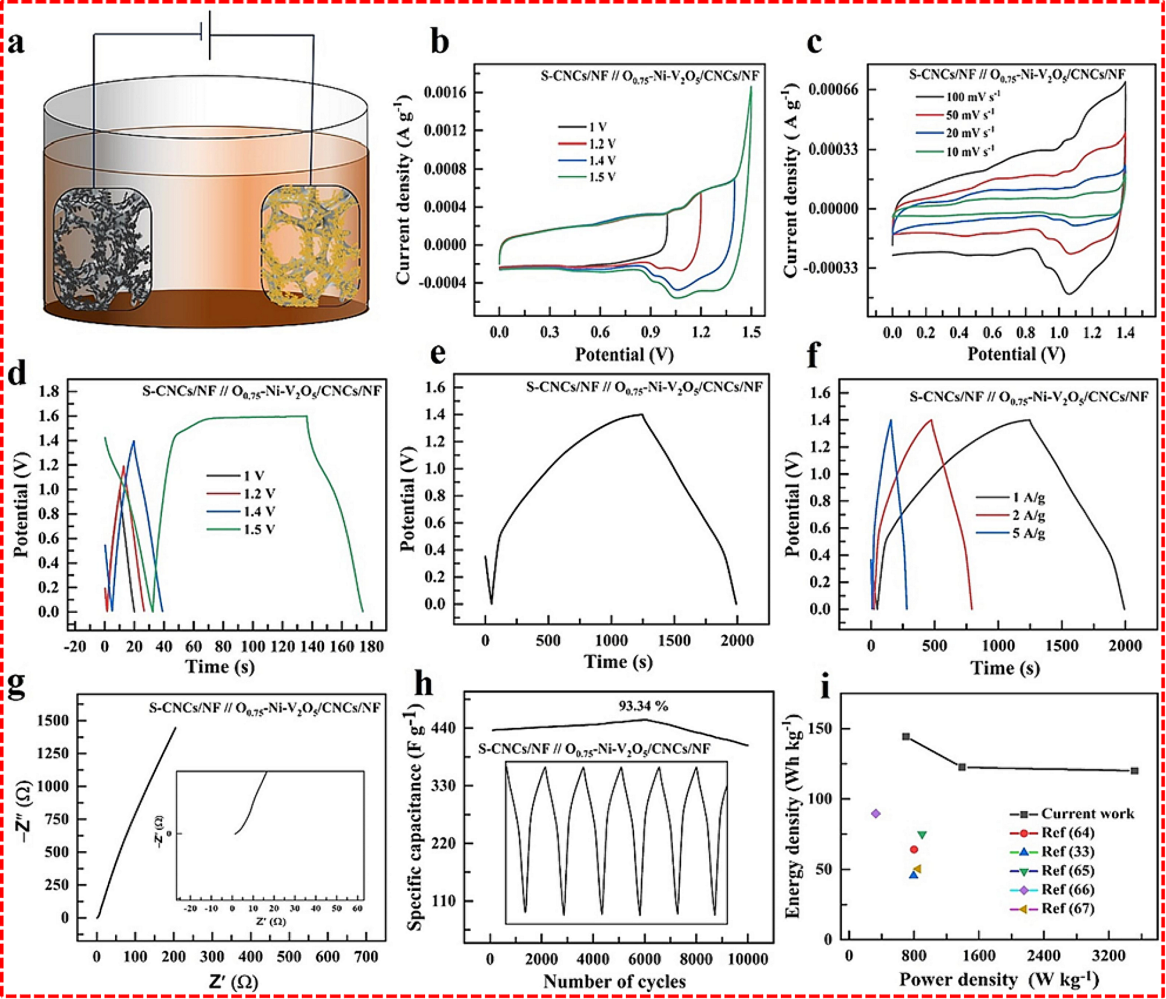
(**a**) Schematic representation of S-CNCs/NF // O_v_-Ni-V_2_O_5_ /CNCs/NF SCs, cyclic voltammetry analysis for (**b**) different potential window, (**c**) various scan rate, Galvanostatic charge-discharge analysis for (**d**) different potential window, (**e**) optimized GCD window, (**f**) different applied current, (**g**) Nyquist plot, (**h**) cyclic stability test, (**i**) Ragone plot [64]

The oxygen-deficient Ni-doped V_2_O_5_ on carbon nanocoils, grown directly on nickel foam (O_v_-Ni-V_2_O_5_ /CNCs/NF), also exhibited improved capacitance behaviors [64]. The synergistic effect of the composite components achieved a maximum specific capacitance of 3485 F/g at a current density of 1 A /g. The full cell was designed using O_v_-Ni-V_2_O_5_ /CNCs/NF as a positive electrode and S-CNCs/NF as a negative electrode, as shown in Fig. 11. The electrochemical study of the device reveals that it operates effectively up to a potential window of 1.5 V, achieving a specific capacitance of 543 F/g at a current density of 1 A/g. Additionally, the performance of this asymmetric SC surpasses that of previously reported systems, as demonstrated by its superior energy and power density metrics. The reported capacitance shows better capacitance than the pure Ni-V_2_O_5_/CNCs/NF.

**Fig. 12:**
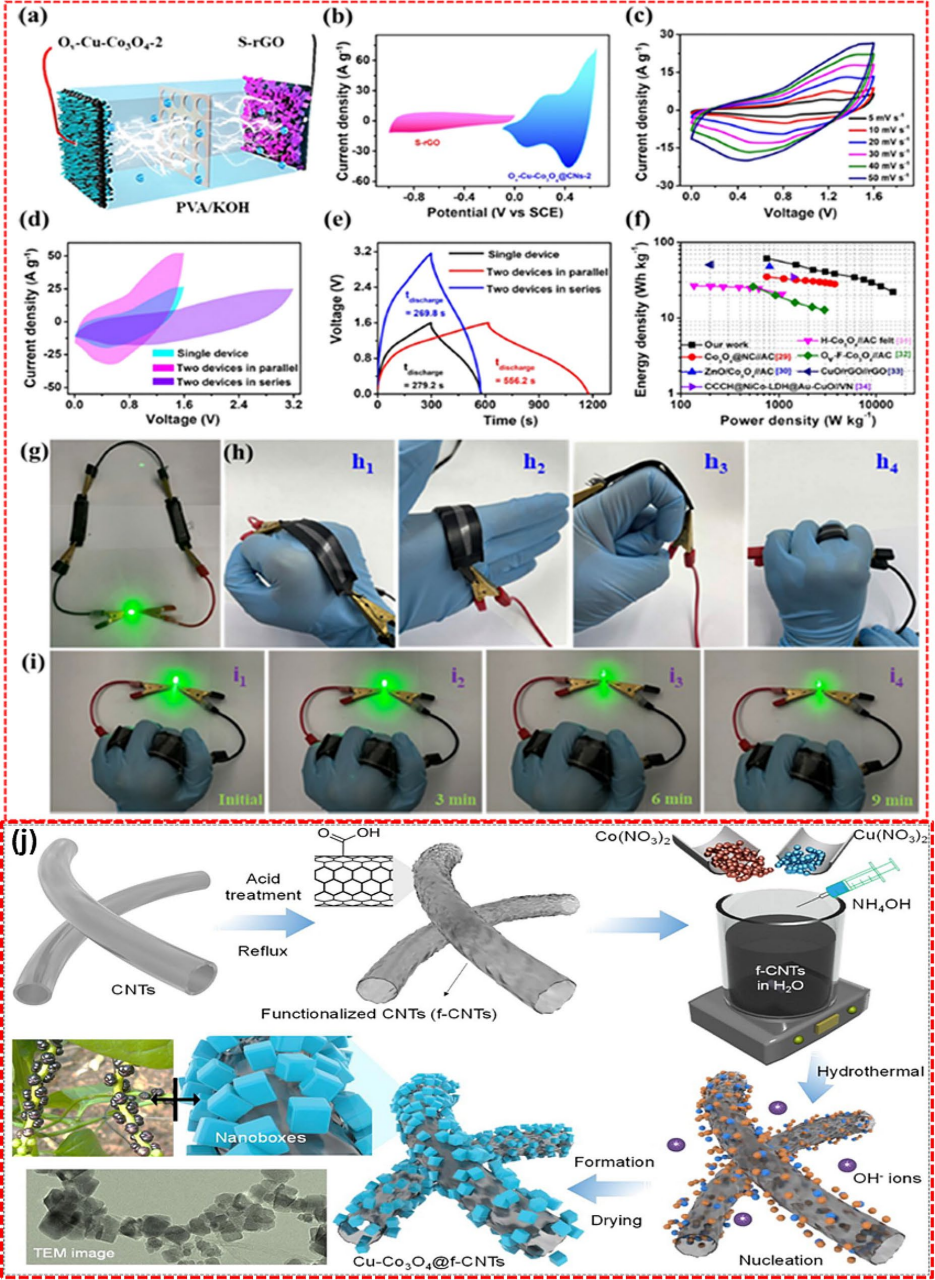
(**a**) Schematic representation O_v_-Cu-Co_3_O_4_@C // S-rGO solid state flexible SC, CV analysis of (**b**) the positive and negative electrodes, (**c**) different scan rates. (**d**-**e**) CV and GCD analysis of a single and two devices connected in parallel and series. (**f**) Ragone plot, (**g**-**i**) practical demonstration of wearable, flexible asymmetric SC by lighting up the green LED. (**j**) Schematic representation of the hydrothermal preparation process of Cu-Co_3_O_4_@f-CNTs. [107, 108]

Furthermore, oxygen-deficient Cu-doped Co_3_O_4_ in a carbon matrix as a battery-type electrode material for the SC application was reported by Liu et al. [107]. Initially, copper-doped cobalt carbonate hydroxide (Cu-CCH) was grown on a carbon cloth substrate, and converted into a Cu-doped cobalt metal-organic framework using 2-methylimidazole. Calcining Cu-doped Co-MOF in an Ar/H_2_ environment to obtain Cu-doped Co_3_O_4_ then embedded into the carbon network. The electrode material was reduced by using NaBH_4_ to produce O_v_-Cu-Co_3_O_4_/C, which achieved the maximum specific capacity of 927 C /g at a current density of 1 A/g. For practical applicability of the electrode material, the full-cell device was fabricated using S-rGO as shown in Fig. 12. The CV and GCD analyses reveal the dynamic electrochemical behaviors and charge-discharge characteristics of the Cu-Co_3_O_4_/C electrodes. Performance comparisons in energy and power density show its superiority over other metal oxide composites. Additionally, the practical application of this wearable, flexible asymmetric SC is demonstrated by its ability to power a green LED, highlighting its functionality [107]. Similarly, Cu-doped Co_3_O_4_/CNT for composite SC application as shown in (Fig. 12(j)) was studied, which nanocomposite exhibits outstanding redox behavior with superior structural stability than the Cu-Co_3_O_4_ [108].

#### Bimetal oxide-based composites for supercapacitor

**Fig. 13:**
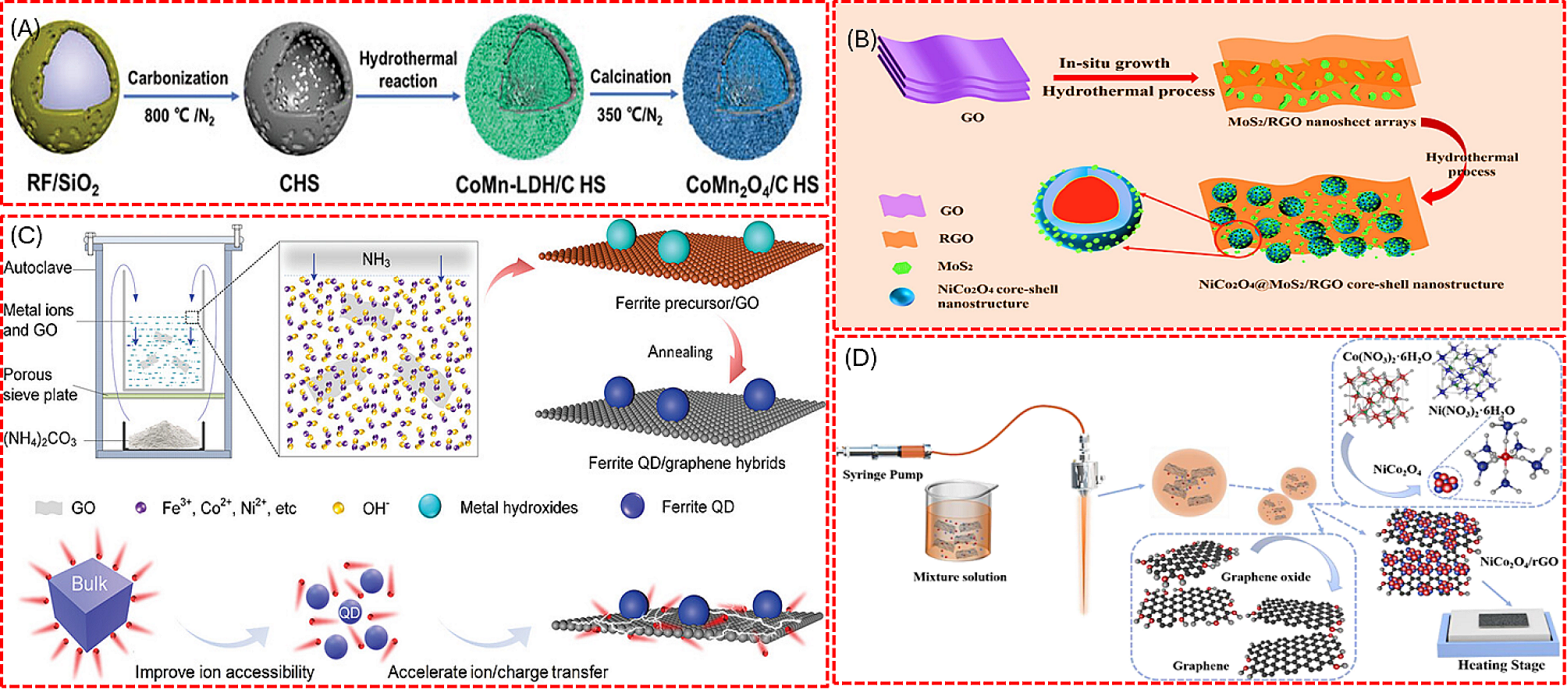
Schematic illustration of hydrothermal preparation of (**A**) CoMn_2_O_4_/C hollow spheres, (**B**) NiCo_2_O_4_@MoS_2_/RGO composite. Schematic representation of preparing (**C**) NiFe_2_O_4_ QD/G nanostructure by biomimetic mineralization synthetic method. (**D**) Binder-free NiCo_2_O_4_/rGO composites by ultrasonic spray method. [109, 110, 112, 113]

In order to further increase the electrochemical performance of metal oxide composites, bimetallic oxides are introduced. The presence of two metal ions can facilitate better electron and ion transport pathways within the electrode material, leading to higher conductivities. In addition, Bimetal oxides often exhibit enhanced structural integrity during the charge-discharge cycles, which helps maintain their shape and functionality over time, thus improving cyclic stability [108]. In this regard, CoMn_2_O_4_/C hollow spheres were utilized as anode material for the sodium hybrid SC. The CoMn_2_O_4_/C hollow spheres were synthesized by a simple hydrothermal reaction followed by calcination (Fig. 13(a)) [109]. Carbon hollow spheres (CHS) with mesoporous structures act as robust, non-sacrificial hard templates. CoMn_2_O_4_ nanosheets are evenly coated over the surface of the CHS, creating a sandwich-like shell configuration. This mesoporous arrangement promotes the infiltration of reactive electrolyte ions, improving their accessibility to the electrode materials. The prepared nanoarchitecture increases the sodium storage ability and reaches the specific capacity of 290 mAh/g at 0.1 A/g. The CoMn_2_O_4_/CHS shows a higher capacity than the CoMn_2_O_4_. The NiCo_2_O_4_/MoS_2_/rGO composite structure, prepared by the hydrothermal method as shown in Fig. 13(b) exhibits the exceptional SC performance [110]. Specifically, the composite demonstrated a remarkable specific capacitance of 946 F/g at 1 A/g, improved rate capability, and outstanding electrochemical stability, maintaining 87.3% retention after 5000 charge-discharge cycles. The study reported the high conductivity of graphene and the high capacitance of NiCo_2_O_4_, is responsible for this superior performance. Furthermore, MXene/NiCo_2_O_4_ composites for asymmetric SC applications was fabricated, reporting an enhanced energy storage performance than the individual Ti_3_C_2_T_x_ and NiCo_2_O_4_ [111].

Ferrite quantum dots (QD) on graphene heterostructures using a simple biomimetic mineralization synthetic method was also introduced, as shown in Fig. 13(c) [112]. The metal precursor, dissolved in deionized water with GO powder, underwent ultrasonication. This mixture was autoclaved, followed by calcination at 500 °C in an N_2_ atmosphere to yield the metal ferrite QD/ graphene. The prepared ferrite QD/ graphene improves electrical conductivity, ion transport, and active sites. The graphene nanosheets support the ferrite QD that is anchored on it. Here, the graphene nanosheets act as a structural material to strengthen the stability, increase charge transfer ability, and enhance the ion diffusion.

The NiFe_2_O_4_ quantum dot/graphene nanostructure exhibits an impressive specific capacitance of 697.5 F/g at a current density of 1 A/g. Meanwhile, the binder-free NiCo_2_O_4_/rGO composite electrode material, produced via an ultrasonic spray method, achieves a higher specific capacitance of 871 F/g at the same current density of 1 A/g [113].

#### Metal oxide composites-based printed supercapacitor

**Fig. 14:**
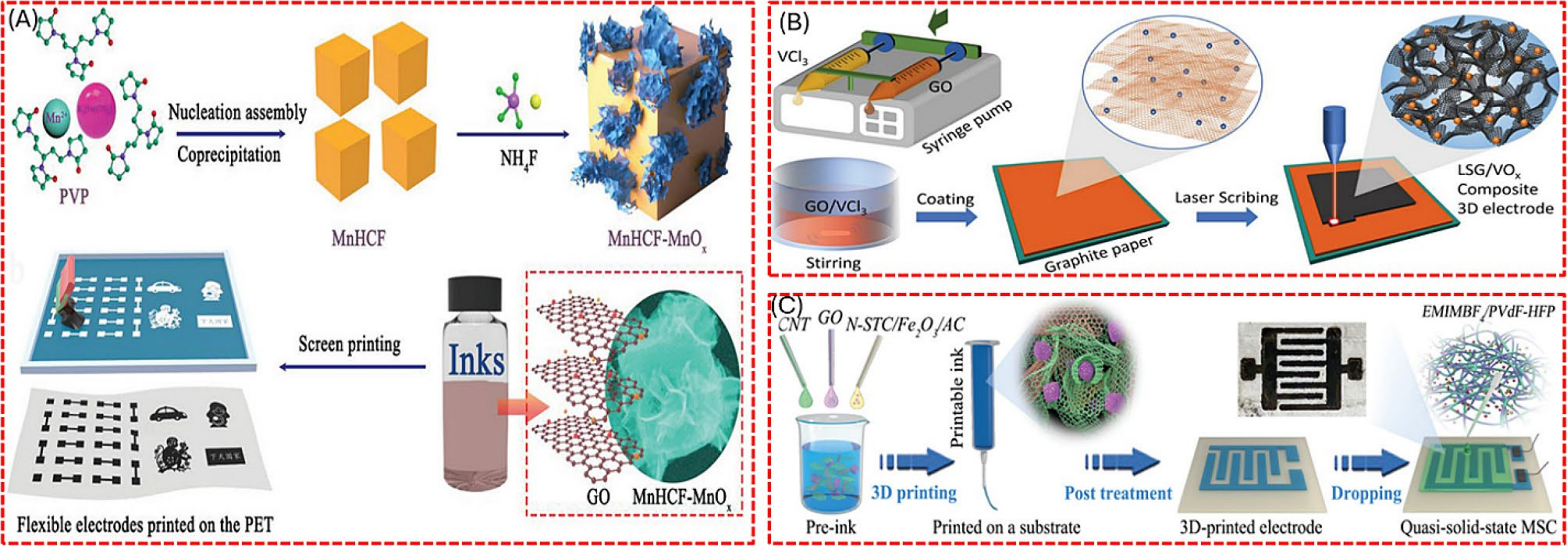
Pictorial representation of preparing (**A**) MnHCF-MnO_x_/ErGO flexible patterned SC by simple screen-printing technique, (**B**) Graphene/VO_x_ flexible symmetric SC by the facile laser-scribing method, (**C**) CNT, GO and N-STC/Fe_2_O_3_/AC active materials micro-mesoporous quasi-solid state micro SC by 3D printing technique. [114–116]

Wearable and flexible low-power electronic devices are attracted to the next revolution of the electronic world. There is a huge demand for onboard energy storage devices that need to be compatible with recent electronic technology. On the way to producing printed, electronic circuit boards, printed SC technology has also boomed nowadays. Liang et al. fabricated a flexible patterned SC using manganese hexacyanoferrate-manganese oxide and electrochemically reduced graphene oxide (MnHCF-MnO_x_/ErGO) as the electrode material by simple screen-printing technique shown in Fig. 14 [114]. MnO_x_ is integrated into the MnHCF system through an in-situ self-reaction with NH_4_F, producing MnHCF- MnO_x_ that delivers a specific capacitance of 467 F/g at 1 A/g. This system also forms a part of a flexible SC, MnHCF- MnO_x_ /ErGO, which achieves an areal capacitance of 16.8 mF/cm². Additionally, a graphene/VO_x_ flexible symmetric SC is developed using a straightforward laser-scribing method. This combination of graphene and vanadium oxides, which feature multiple oxidation states, not only broadens the potential window but also enhances the specific capacitance to 1110 F/g. Notably, the gravimetric capacitance of graphene/VO_x_ significantly surpasses that of a bare graphene electrode [115].

In addition, a 3D printed micro-mesoporous quasi-solid state micro SC was demonstrated by Lai’s group, as shown in Fig. 14(c). The electrode is prepared as an ink by combining active materials such as CNT, GO, and N-STC/Fe_2_O_3_/AC. Once printed, the electrode material is densely decorated with ultrafine Fe_2_O_3_ nanoparticles. This 3D-printed electrode, when paired with an ionogel electrolyte, demonstrates a gravimetric capacitance of 267 F/g at a scan rate of 2 mV/s. Notably, the N-STC/Fe_2_O_3_ nanocomposites exhibit superior capacitance compared to each of the individual components within the composite [116].

### Metal oxide composites for battery

Metal oxide nanocomposites are pivotal in propelling battery technology forward, acting as essential elements within the energy storage mechanisms of various battery systems. Their application spans lithium-ion (LIB), sodium-ion (NIB), Zinc-ion (ZIB), potassium-ion (KIB), and lithium-sulfur (Li-S) batteries, driving significant advancements in energy storage capabilities [117–119]. The dominance of metal oxide composites in battery technology stems from their high surface area, superior electrochemical performance, enhanced ion diffusion rates, adjustable properties, and seamless compatibility with electrolytes, marking them as indispensable in the evolution of battery technologies.

In particular, catalytic activity is crucial and significantly boosts electrochemical performance. Metal oxide composites, blending the catalytic traits of metal oxides with carbon-based materials or catalytic metals, exhibit synergistic effects that amplify catalysis [120]. These composites, with their distinctive surface attributes, electronic structures, and reactivity, emerge as potent catalysts for diverse chemical reactions. Incorporating graphene or CNTs increases active sites and enhances electron transfer, improving catalysis [26].

#### Anodes

**Fig. 15:**
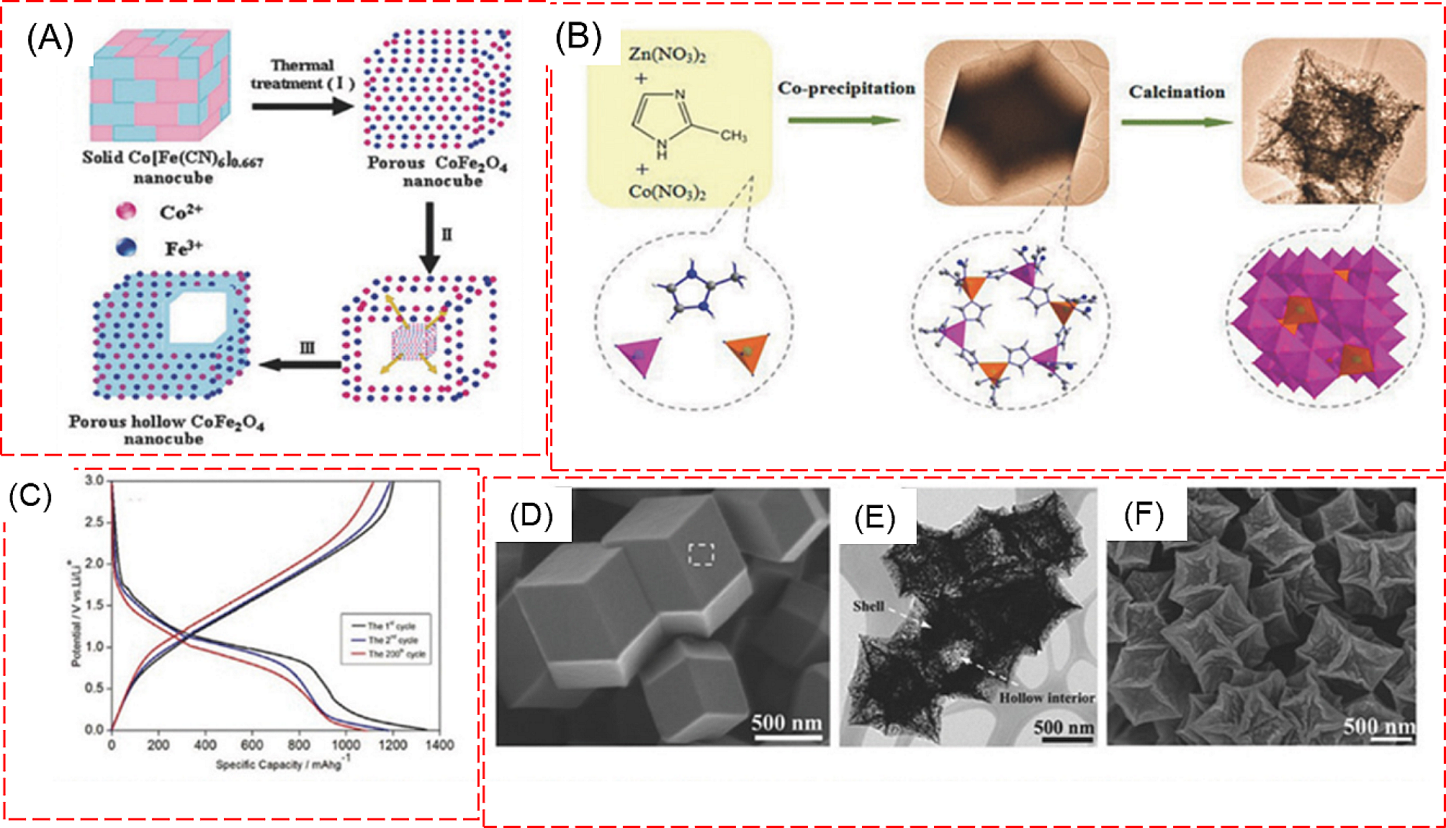
(**A**, **B**) Schematic drawing illustrating the preparation CoFe_2_O_4_ and Zn-Co-ZIFs-0.33 (**C**) specific capacity analysis of CoFe_2_O_4_. (**D**, **E**, **F**) FESEM images and TEM images of ZnxCo_3_xO_4_ hollow polyhedral obtained by annealing Zn-Co-ZIFs-0.33 [121, 122]

The diverse approaches and technological advancements in synthesizing and applying metal oxide composites for energy storage highlight their crucial role as anodes in batteries [120–123]. Nanocomposites in battery anodes help buffer volume changes during charge/discharge cycles, reducing material stress and enhancing cycling stability. The controllable conductivity enhance high-rate performance. Additionally, their robust structures maintain integrity under mechanical stresses, prolonging battery life. The hollow porous CoFe_2_O_4_ nanotube anode from metal–organic frameworks (MOF) was prepared, whose schematic representation of synthesis is shown in Fig. 15 [121]. The CoFe_2_O_4_ was used as an anode for LIB and its stable capacity arrives at 815 mA h/g at 20 ºC. By adjusting synthesis parameters, the surface area, morphology, and chemistry of composites are finely tuned to enhance the number of active sites and optimize interactions with reactants for improved catalytic efficiency, as evidenced by charge-discharge analysis. A detailed morphological analysis reveals Zn_x_Co_3-x_O_4_ hollow polyhedral structures at the nanoscale, exposing their internal hollow configurations [122]. Additionally, TEM imaging offers a deep dive into the crystal lattice, grain boundaries, and material defects, providing a richer understanding of the metal oxides and their composites. Such insights into atomic arrangement, phase purity, and crystallinity elucidate their diverse electrochemical properties, highlighting their significance in developing advanced energy storage devices with practical applications.

A novel TiO_2_ decorated MXene composite as LIB anodes was also reported. The limitations of TiO_2_, such as low electronic conductivity, were overcome by leveraging the excellent structural stability and superior electronic conductivities of MXenes. Thus, electrochemical tests demonstrated that MXene- TiO_2_ nanocomposite anode had faster kinetics and significantly better performance, including a higher capacity of around 200 mAh/g at 0.1 C, compared to pristine TiO_2_ anodes [123].

Additionally, extensive research has been conducted on graphene and GO composites. Synthesizing these metal oxides on graphene in various morphologies including nanosheets, nanorods, and complex hierarchical structures has led to improved electrochemical behavior. These structures increase the surface area and offer more active sites for lithium interactions while also shortening the pathways for electron and ion movement, contributing to superior battery performance [124]. V_2_O_3_ nanoparticles on nitrogen-doped carbon nanosheet arrays in Ni foam were synthesized as anode for LIB, paired with a Li metal foil cathode. This electrode displayed high initial capacities of 984 mAh/g (discharge) and 508 mAh/g (charge), maintaining a capacity of 317 mAh/g after 1000 cycles at 2 A/g. The improved performance, due to increased conductivity and reduced volume expansion, is credited to the nitrogen-doped carbon coating and the structural stability from the tight contact with the Ni foam [125]. On the other hand, Tin oxide (SnO_2_) nanoparticles anchored on graphene sheets offer a high specific capacity attributed to the reversible alloying/dealloying reactions of tin with lithium ions, as well as the high surface area of graphene for lithium-ion adsorption. Tin oxide/graphene composites typically exhibit specific capacities ranging from 600 mAh/g to 1000 mAh/g [126]. The specific capacity of tin oxide electrodes is around 700 mAh/g, while the graphene component contributes negligibly to the capacity due to its low lithium storage capability.

**Fig. 16:**
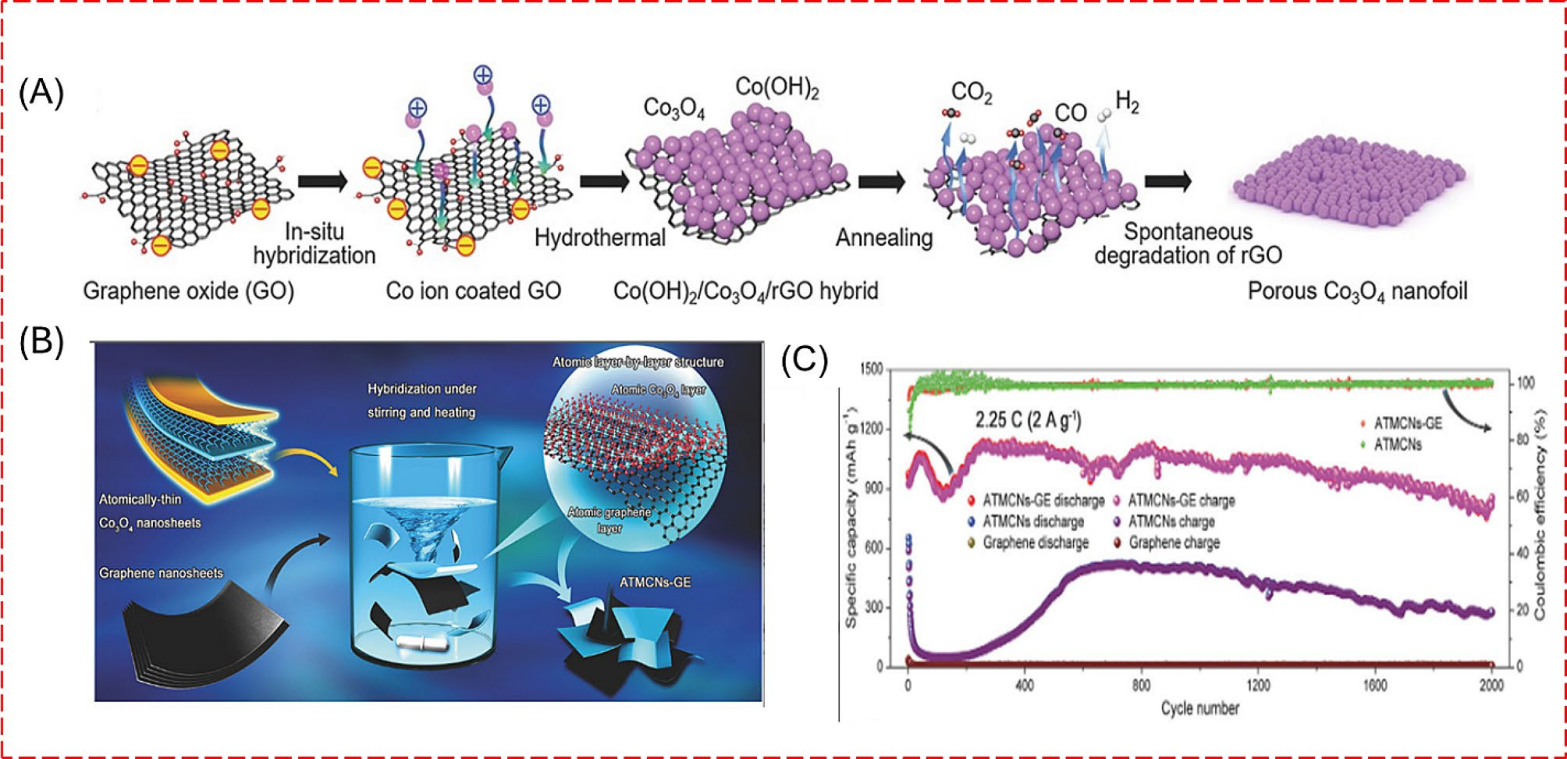
(**A**, **B**) illustrates a novel synthesis method for 2D porous Co_3_O_4_ nanofoils using GO as a sacrificial template. This method leverages the thermal instability of GO and the catalytic ability of Co_3_O_4_ particles to fabricate the nanofoils, aiming to enhance the performance of lithium-ion battery anodes. (**C**) Cyclic stability comparison of nanocomposite and graphene measured at a high current density of 2.25 C [127, 128]

A 2D Co_3_O_4_ nanofoils using GO as a sacrificial template as shown in the Fig. 16 (a) was prepared by hydrothermal method. The synthesized nano foils exhibit a high reversible capacity of 1279.2 mAh/g, after 50 cycles, which represents the significant conversion mechanism from Co_3_O_4_ to Li_2_O and metallic Co [127]. An atomic layer-by-layer Co_3_O_4_/Graphene Composite was synthesized by Dou et al. as shown in Fig. 16 (b) and the stability analysis was reported along with the comparison of atomically thin mesoporous Co_3_O_4_ nanosheets (ATMCNs) and its graphene composite as shown in Fig. 16 (c) [128].

Furthermore, mesoporous ZnCo_2_O_4_ microspheres composed of ultrathin nanosheets cross-linked with metallic NiSi_*x*_ nanowires on Ni foam were utilized as anodes for LIB [129]. This composite exhibits high specific capacity, excellent rate capability, and long-term cycling stability when used as an electrode material in LIB. The hierarchical structure provides abundant active sites for lithium-ion storage, while the NiSi_x_ enhances electron conductivity and structural stability.

#### Cathodes

Metal oxide composites are indispensable as cathode materials in batteries due to their diverse advantages by offering high energy density, enabling batteries to store substantial amounts of energy efficiently. Moreover, they enhance the stability of cathodes, mitigating issues of structural degradation during charge-discharge cycles, thus prolonging battery lifespan. By incorporating conductive additives, metal oxide composites may improve electron transport within the cathode, optimizing battery performance. Additionally, they facilitate ion diffusion, enabling faster charging/discharging rates, which is crucial for demanding battery applications like electric vehicles and portable electronics. Metal oxide composites serve as crucial cathode materials in LIB, NIB, and ZIB, maintaining high performance over numerous charge-discharge cycles.

**Fig. 17:**
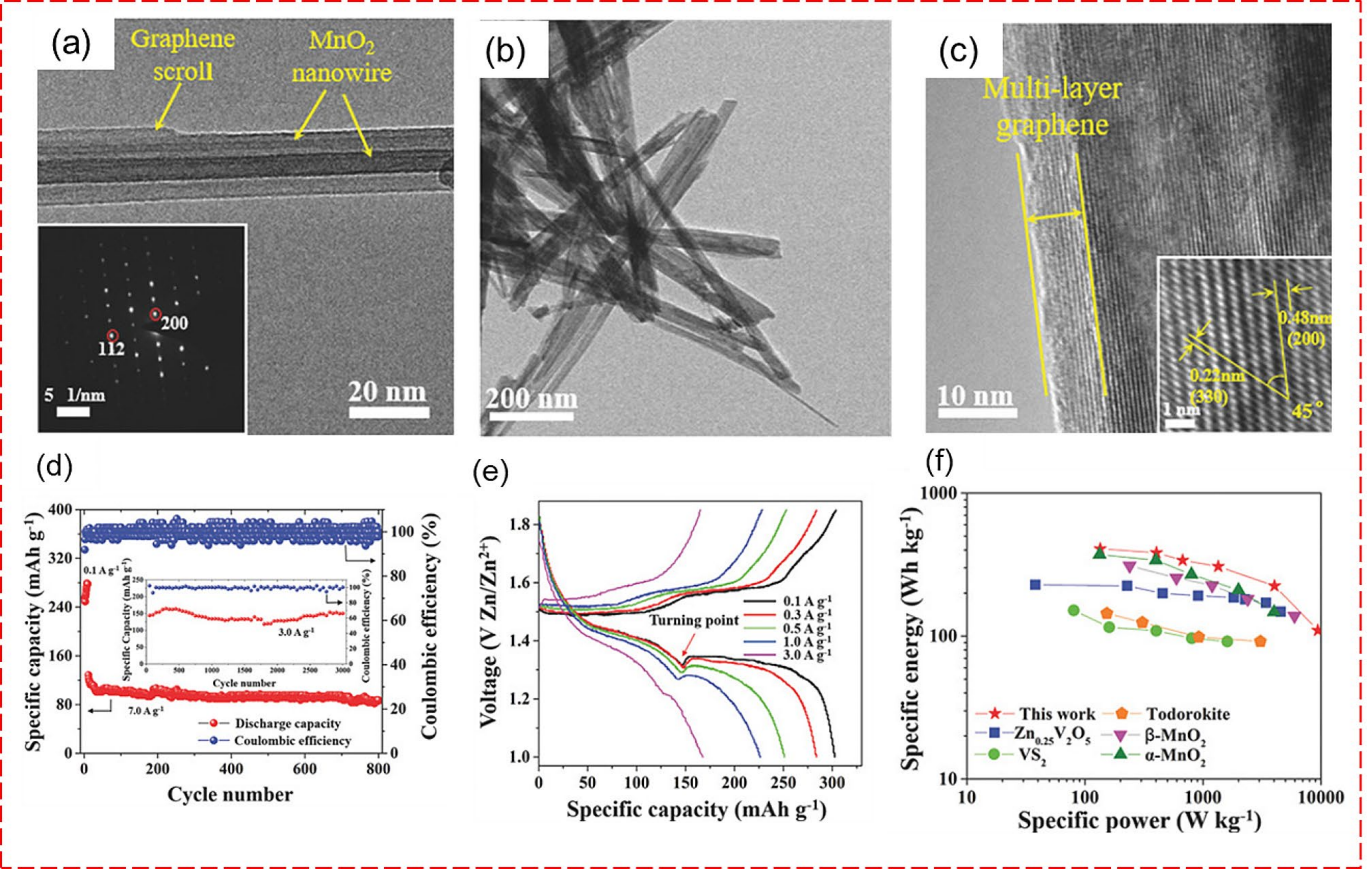
(**a**-**c**) TEM images of α-MnO_2_ nanowires with graphene scrolls. (**d**) stability analysis for 3000 cycles (**e**) GCD analysis at different current densities. (**f**) Ragone plot comparison of α-MnO_2_ Nanowires with graphene scrolls [145]

Along the way of developing effective cathode materials, graphene-coated α-MnO_2_ nanowires for high-performance cathode material for aqueous ZIB was prepared by facile hydrothermal method, as shown in Fig. 17. The stability of the cathode material was enhanced by incorporating rGO, as evidenced by a high capacitance retention of 94% after 3000 cycles. The addition of rGO minimized the dissolution of the cathode and boosted its electrical conductivity, leading to a high capacity of 362.2 mA h/g at a current density of 0.3 A/g after 100 cycles [110]. To further enhance the stability of ZIB, nitrogen was doped into MnO_2_. In this study, the stability of ZIB was evaluated at a current density of 5 A/g for both MnO_2_ and nitrogen-doped MnO_2_ cathodes. The capacitance retention improved from 62 to 83% with the introduction of nitrogen into MnO_2_ [130].

In the view of bimetallic transition metal oxide composites, a nonstoichiometric ZnMn_2_O_4_/carbon composite was employed as a new Zn-insertion cathode material-based aqueous battery with a reversible capacity of 150 mAh /g and a capacity retention of 94% over 500 cycles. The high performance was credited to the remarkable cathode performance from the facile charge transfer and Zn insertion in the structurally robust spinel featuring small particle size and abundant cation vacancies [131]. A high-performance Zn-air battery was reported using Ni-doped Co oxide nanosheets on carbon-fiber paper as an air cathode. This cathode, coupled with a Zn foil anode and 6 M KOH electrolyte, delivered a specific capacity of 655 mAh/g at 30 mA/cm² and achieved a power density of 377 mW/cm², outperforming traditional CoO nanosheets and Pt/C catalysts by 20% [132].

Moreover, complex metal composites, including Al_2_O_3_ coated LiNi_0·5_Mn_1·5_O_4_ by the solid-state reaction by atomic layer deposition, reported better cyclic stability with capacity retention over 63% after 900 cycles. Note that the bare LiNi_0·5_Mn_1·5_O_4_ can only maintain 75% after 200 cycles. The study also reported temperature-dependent changes in specific capacity where the Al_2_O_3_ coated LiNi_0·5_Mn_1·5_O_4_ delivered 116 mAh/g at a higher temperature of 55 °C whereas the bare LiNi_0·5_Mn_1·5_O_4_ reduced to 98 mAh/g after 100 cycles respectively [133]. Furthermore, a LIB was constructed using a Li_2_ZrO_3_ coated LiNi_0·7_Co_0·15_Mn_0·15_O_2_ composite cathode paired with a graphite anode, fabricated through the precipitation method. The electrochemical performance of this setup was thoroughly examined, revealing that the composite cathode maintained 73.3% capacity retention even after 1500 cycles at a current rate of C/3 [134].

A thin film of LiCoO_2_ cathodes using aluminum oxide coating to suppress the cobalt dissolution from the cathode using different metal oxides was examined. The study concluded that ZrO_2_-coated LiCoO_2_ could efficiently inhibit cobalt dissolution, resulting in an excellent electrochemical behavior above 4.4 V [135]. Multiple metal oxides (Co_3_O_4_/rGO, Fe_2_O_3_/rGO, and CoFe_2_O_3_/rGO) as cathode materials using Prussian blue analog (PBA)-type MOF (CoCo(CN)_6_, FeFe(CN)_6_, and FeCo(CN)_6_) as precursors for AIB were established with profound significance. Amongst, CoFe_2_O_4_/rGO exhibits a highly stable charge/discharge with a capacity retention rate of 74% after 500 cycles and promising Coulombic efficiency of 99.6% [136]. Aside from carbon-based composite materials, cobalt oxide/phosphate (Co_3_O_4_/CoP) composite reported high-performance Li/S cathode electrocatalyst. The Co_3_O_4_/CoP composite cathode offered high cyclic stability with a very less capacity fading rate of 0.033% per cycle at 1 C rate over 500 cycles [137].

**Fig. 18:**
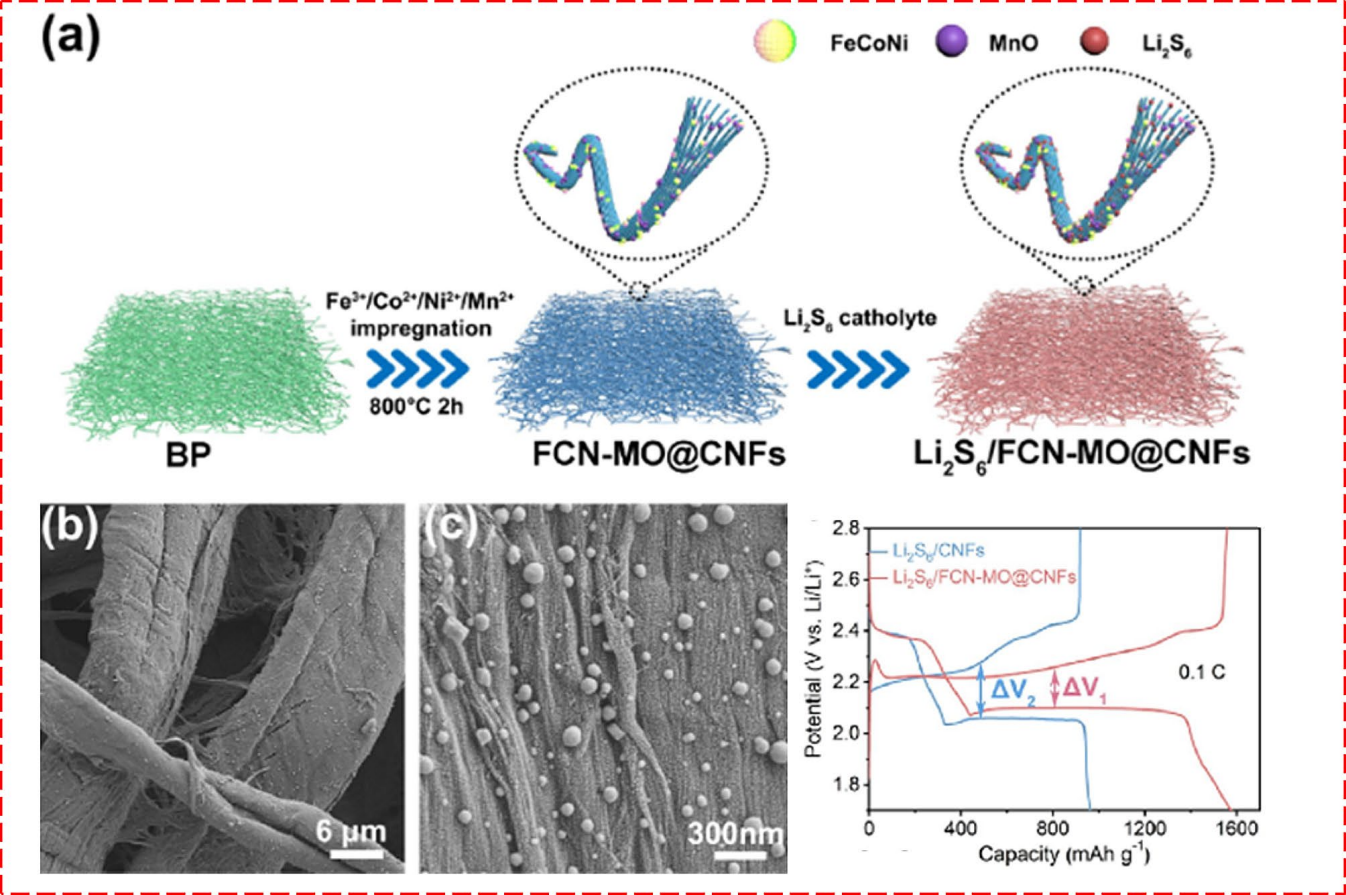
(**a**) The synthesis process of Li_2_S_6_/FCN-MO@CNFs.(**b**-**c**) SEM images of FCN-MO@CNFs and (**d**) Constant-current discharge/charge curves of Li_2_S_6_/CNFs and Li_2_S_6_/FCN-MO@CNFs [139]

The mechanism of thermal decomposition of the cathode was carefully explored using high-nickel (LiNi_0.87_Co_0.05_Mn_0.08_O_2_) composites with loaded nano-Al_2_O_3_ on conductive carbon for LIBs. The study demonstrates a significant enhancement in the thermal properties of delithiated composite cathodes when paired with electrolytes, showcasing a remarkable 44% reduction in maximum heat flow, decreasing from 5.59 mW m/g to 3.15 mW m/g. Furthermore, the phase transition from spinel to rock salt is noticeably delayed while maintaining comparable electrochemical performance [138]. A nano-seized uniformly compounded FeCoNi and MnO nanoparticles for the low-temperature cycling performance of Li-S batteries is shown in Fig. 18 (a-c). The addition of MnO nanoparticles enhances absorption, and acting as anchors for Li polysulfides by increasing their local concentration around both FeCoNi and MnO nanoparticles [139]. This effectively reduces the shuttle effect and improves cycling stability, especially at low temperatures. At -40 °C and a rate of 0.1 C, the initial discharge capacity reaches 1167.5 mAh/g, maintaining 70.1% capacity after 100 cycles at 0.2 C and its capacity is compared with the composite material as shown in Fig. 18 (d). This study offered a novel strategy for developing high-performance, low-temperature Li-S batteries.

**Fig. 19:**
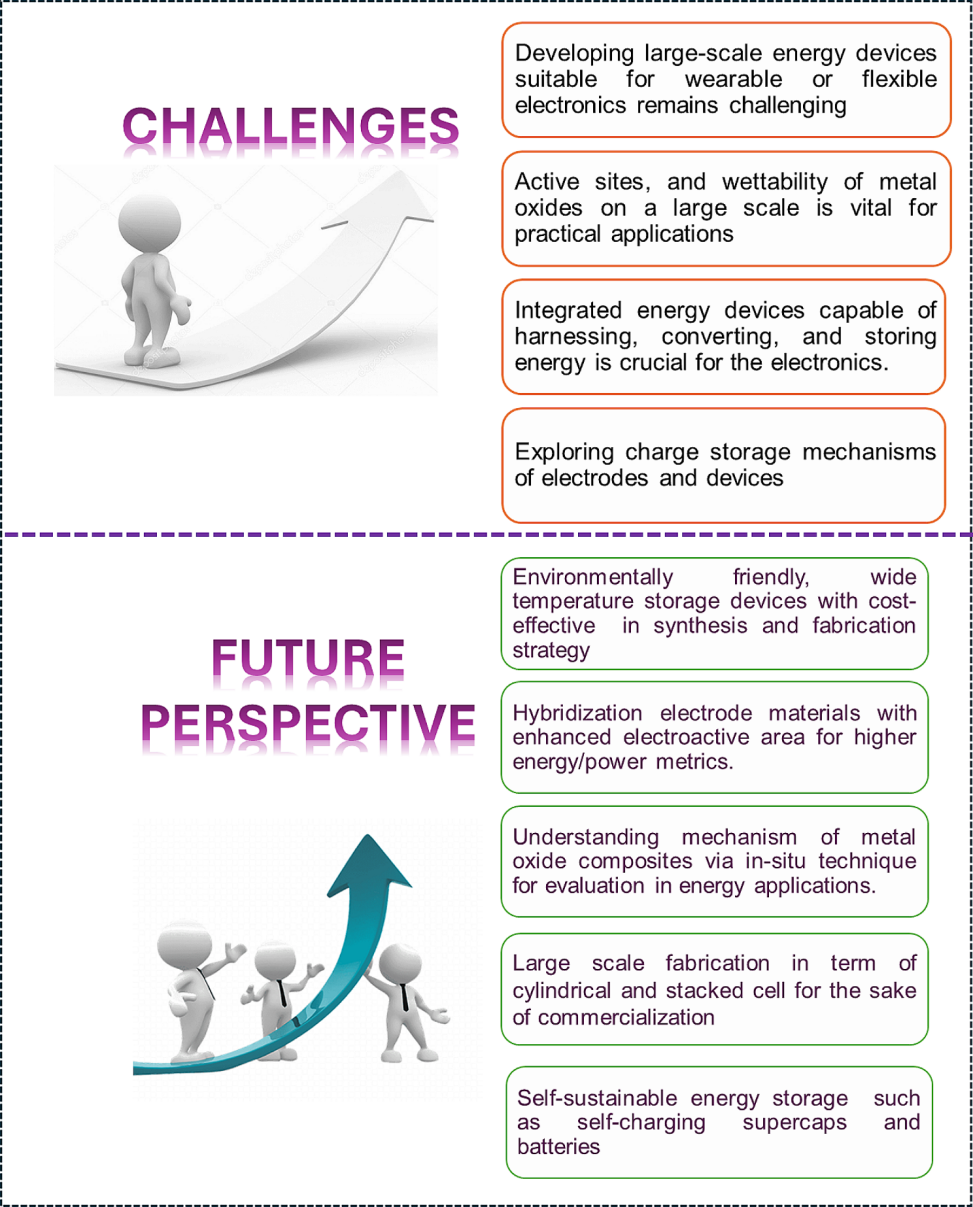
Challenges and future perspectives for the energy storage devices

Through a simple dry ball-milling method, O3-type Na[Ni_0.6_Co_0.2_Mn_0.2_]O_2_ cathode was synthesized, and Al_2_O_3_ nanoparticle was coated over the cathode material, which provides a high energy density for NIB. The Al_2_O_3_-coated Na[Ni_0.6_Co_0.2_Mn_0.2_]O_2_ cathode demonstrated exceptional performance, boasting a high specific capacity of 151 mA h/g, coupled with enhanced cycling stability and rate capability in a half-cell configuration. Additionally, this cathode innovation was successfully scaled up to a pouch-type full cell, employing a hard carbon anode. Notably, the full cell exhibited superior rate capability and remarkable capacity retention, maintaining 75% of its capacity after 300 cycles. With an impressive energy density of 130 W h/kg, this advancement underscores the potential of Al_2_O_3_-coated cathodes and hard carbon anodes in pushing the boundaries of battery technology towards more efficient and sustainable energy storage solutions [140].

The significance of metal oxide composites in electrochemical energy storage systems has been thoroughly demonstrated, showcasing their specific capacity, cycle life, and rate capability. However, to develop high-performance materials for future energy applications, several critical factors must be considered and addressed.

## Factors for consideration for designing metal oxide composites

For optimal electrochemical energy storage, electrode materials should exhibit several key characteristics: (i) a large specific surface area to provide abundant electroactive sites, (ii) high electrical conductivity for fast charging and discharging, (iii) robust electrochemical stability, (iv) a broad operational temperature range for functionality under extreme conditions, (v) a wide operating voltage window suitable for both aqueous and ionic electrolytes, and (vi) cost-effectiveness to enable scaling from laboratory to industrial production.

In this review article, we explore the preparation methodologies and various configurations of metal oxide composites for electrodes of energy storage devices, including supercapacitors (SCs) and batteries. Currently, energy storage devices face challenges such as suboptimal energy/power ratings and limited cycle life. Addressing these issues requires focused research on developing diverse strategies to enhance system outcomes through innovative electrode material design, which is essential for advancing the performance and longevity of energy storage technologies.

Designing low-dimensional nanostructured electrode materials with high surface areas and differentiated morphologies is crucial for enhancing the electrochemical performance of these devices. Especially for SCs, the potential limit can significantly vary depending on the electrolyte system. Therefore, designing electrodes that work effectively across various electrolyte types and can operate at higher potential voltages is crucial for achieving high-performance devices. Additionally, designing electrodes with enhanced flexibility is vital for their application in flexible electronics.

Moreover, multi-functionality in energy materials can enhance their attractiveness in energy storage applications. These functionalities could include integration into electrochromic devices, AC line-filtering, and the development of self-healing, shape-memory, and self-charging energy cells, each feature adding value and expanding the potential applications and effectiveness of energy storage devices.

For commercialization, it is crucial to consider other complex factors beyond the basic functionality of the product. Self-discharge is a significant limitation of energy storage devices, hindering their practicability. In batteries, the self-discharge behavior is minimal, whereas in SCs, it is high, complicating their use in real-time applications due to rapid energy loss over time. Enhancements in electrode materials, electrolytes, and separators, such as adopting solid electrolytes and innovative membranes with sulfonated functional groups, are crucial to mitigate self-discharge rates.

Furthermore, optimizing performance metrics is key to improving energy efficiency, cycle life, and cost-effectiveness. Developing scalable manufacturing processes that streamline production and reduce costs is vital for the economic viability of these devices at an industrial scale. Additionally, adhering to regulatory standards is crucial as they provide clear frameworks for performance, safety, and environmental impact, thereby playing a pivotal role in the broader market adoption of energy storage technologies.

## Conclusion and future perspectives

In this review, we have examined the role of metal oxide composites as electrodes in energy storage applications, focusing on SCs and batteries with varying architectures and designs. We discussed various methodologies for preparing metal oxides and their composite derivatives, emphasizing the integration of diverse components. For optimal energy system development, electrode materials should have a high surface area, numerous active sites for electrochemical charging/discharging, and a specific surface morphology to minimize ion diffusion length. The schematics for the challenges and the future perspectives of the metal oxides composites for the energy storage applications is provided in Fig. 19.

Despite significant research efforts in engineering metal oxide composites, there remain substantial challenges to developing efficient systems. The future direction of existing research technologies includes:

i) The development of large-scale energy devices suitable for wearable or flexible electronics remains a formidable challenge. It is also essential to devise environmentally friendly, low-temperature, and cost-effective synthesis methods. Additionally, enhancing the surface area, active sites, and wettability of metal oxides on a large scale is vital for practical applications.
ii) Improving the conductivity of metal oxides through hybridization with carbonaceous materials and other electroactive substances can lead to synergistic effects. These enhancements can increase surface area, create porosity, facilitate electron/proton conduction, and add pseudocapacitance.
iii) Designing integrated energy devices capable of harnessing, converting, and storing energy is crucial for the electronics. Self-charging energy devices, particularly using metal oxides with their high performance and tunable characteristics, show promise for powering portable, and implantable electronics, though research in this area is still in its early stages.
iv) Exploring the in-depth charge/storage mechanisms of electrodes/devices through operando spectroscopic studies and theoretical investigations is an emerging research area. Understanding the roles of modified metal oxide-based materials in energy applications can significantly enhance energy storage performance.
v) Exploring the hybridization of metal oxides with emerging low-dimensional materials such as MXenes, black phosphorus, and transition metal dichalcogenides promises high-performance energy storage devices. Determining the optimal ratios and combinations of these materials with metal oxides is crucial for developing superior electrode materials.

In summary, while recent developments and strategies in metal oxides and their composites for energy storage devices show promise, there is a continuing need for further research to transition these technologies to industrial-scale applications effectively. This review underscores both the current achievements and the considerable potential for future advancements in this rapidly evolving field.

## Data Availability

The datasets used and/or analyzed during the current study are available from the corresponding author upon reasonable request.
